# Cross-country health inequality in the asthma burden: findings from the global burden of disease study 2021

**DOI:** 10.1186/s12889-025-25149-y

**Published:** 2025-11-17

**Authors:** Siying Zhang, Yumei Zhong, Yingjie Teng, Lijun Tang, Yun Zhou, Wenge Li, Zongshi Gao, Hui Gao, Fang-biao Tao, Xiulong Wu

**Affiliations:** 1 Department of Maternal, Child and Adolescent Health, School of Public Health, Anhui Medical University, No 81 Meishan Road, Hefei, Anhui 230032 China; 2 Key Laboratory of Population Health Across Life Cycle (Anhui Medical University), Ministry of Education of the People’s Republic of China, No 81 Meishan Road, Hefei, Anhui 230032 China; 3 Anhui Provincial Key Laboratory of Environment and Population Health across the Life Course, Anhui Medical University, No 81 Meishan Road, Hefei, Anhui 230032 China; 4 The First Clinical college of Anhui Medical University, Anhui Medical University, No 81 Meishan Road, Hefei, Anhui 230032 China; 5 Department of Pediatrics, The First Affiliated Hospital of Anhui Medical University, No 218 Jixi Road, Hefei, Anhui 230022 China; 6 Wujing Community Healthcare Service Center of Minhang District, Shanghai, 201100 China

**Keywords:** Asthma, Health inequality, Smoking, Body mass index, Occupational exposure

## Abstract

**Background:**

Changes in inequality in the total and risk factor-specific incidence, prevalence, deaths, and disability-adjusted life years (DALYs) of asthma have not been evaluated.

**Methods:**

Asthma incidence, prevalence, deaths, and DALYs, and risk factors-related deaths and DALYs rates were extracted from the Global Burden of Disease study 2021. We employed decomposition analysis, encompassing aging, population growth, and epidemiologic changes, to investigate factors influencing incidence, prevalence, deaths, and DALYs of asthma. Besides, the slope inequality index (SII) and relative concentration index (RCI) were used to assess the inequality in the total and risk factor-specific incidence, prevalence, deaths, and DALYs of asthma.

**Results:**

Global rates of asthma incidence, prevalence, deaths, and DALYs declined from 1990 to 2021. People aged < 5 years exhibited the highest asthma incidence, and those aged ≥ 70 years old had the highest prevalence, death, and DALYs rates of asthma. Leading drivers of increased number of asthma deaths were aging (127.48%) and population growth (138.99%), but epidemiological change contributed mostly to decreased numbers of asthma incidence, prevalence, and DALYs. Asthma incidence decreased with raising socio-demographic index (SDI), but asthma prevalence increased with increasing SDI. Inequality in asthma incidence and prevalence decreased as indicated by SII and RCI. Besides, asthma deaths and DALYs rates were concentrated in lower SDI regions. Inequality in high body mass index-related asthma death increased and concentrated in lower SDI regions, but inequality in risk factor-specific asthma DALYs all decreased.

**Conclusion:**

Our findings imply that rates of asthma incidence, prevalence, deaths, and DALYs decreased, but age-specific distribution was not essentially changed. Inequality in asthma incidence and prevalence narrowed, but high body mass index-related asthma death concentrated in lower SDI regions and inequality widened. Healthcare system reforms should focus on primary risk factor and lower SDI regions.

**Supplementary Information:**

The online version contains supplementary material available at 10.1186/s12889-025-25149-y.

## Take-home message

While global asthma burden decreased during 1990–2021, persistent inequalities emerged: the high SDI region showed higher asthma prevalence, the low SDI region faced higher incidence, death, and DALYs, demanding tobacco control, obesity intervention, and occupational protection.

## Introduction

Asthma is a chronic respiratory disease characterized by variable airflow obstruction, airway inflammation, and hypersensitivity [1, 2]. In 2019, the Global Burden of Disease (GBD) study reported that age-standardized prevalence, deaths, and disability-adjusted life years (DALYs) rates for asthma were 3,415.5/100,000, 5.8/100,000, and 273.6/100,000 worldwide, respectively [3]. According to global asthma reports, the global prevalence of asthma was 9.1% in children, 11.0% in adolescents, and 6.6% in adults [4]. Asthma affects all age groups and both sexes, so it is necessary to dynamically monitor age- and sex-specific secular trends of asthma incidence, prevalence, deaths, and DALYs. What’s more, demographic expansion and epidemiologic transition have significantly influenced the epidemiology of non-communicable diseases, including asthma [5]. Decomposition analysis would help to reveal the leading drivers of asthma incidence, prevalence, deaths, and DALYs.

Notably, asthma burden (including incidence, prevalence, death, and DALYs) exhibits geographic and socioeconomic disparities. Furthermore, childhood asthma prevalence was higher in developed countries than in developing countries [6, 7], asthma deaths rate was higher in low- and middle-income countries [3]. Modifiable risk factors, including environmental and occupational exposures and behavior factors, play a central role in the development and progression of asthma [8, 9]. According to the GBD 2021 study, smoking, high body mass index (BMI), and occupational asthmagens contributed 9.9%, 16.9%, and 8.8% to global asthma DALYs, respectively [3]. High socio-demographic index (SDI) countries have effectively implemented strict tobacco control measures, while low SDI countries have witnessed a significant increase in tobacco industry activity, resulting in a rapid surge in smoking rates [10]. Furthermore, obesity can potentially lead to changes in respiratory mechanics, including increased respiratory effort and decreased respiratory muscle strength. Obesity prevalence was rising in developing countries [11], along with the persistent high prevalence of obesity in developed countries [12]. The respiratory symptoms of occupational asthma cases may deteriorate in approximately 25%−50% of people due to exposure to asthma-inducing agents in the workplace [13]. In developing regions like Africa and South Asia, occupational asthma is the second most prevalent lung disease [14], accompanied by high rates of deaths and DALYs caused by occupational asthmagens-related asthma. Major epidemiological shifts have taken place in these three attributable risk factors for asthma over the past 30 years, which may lead to further changes in the pattern of health inequality in the asthma burden.

Many studies have explored the changing pattern of asthma epidemiology from 1990 to 2021^3,14^; however, no study has quantified the global disparities in asthma burden across countries with different SDI levels from 1990 to 2021. Compared to other inequality measures (e.g., Gini coefficient), the slope inequality index (SII, representing absolute inequality) and the relative concentration index (RCI, indicating relative inequality) are ordered measures of social inequality. These metrics can be used to evaluate how asthma incidence, prevalence, deaths, and DALYs vary with increasing SDI [15]. Besides, the risk factors attributable to social inequalities were also unknown.

Our study primarily aimed to explore global health inequality in asthma burden, and enhance current understanding of sex- and risk factor-specific disparities in asthma incidence, prevalence, deaths, and DALYs rate. Additionally, we also performed trend and decomposition analysis of asthma burden. Measuring global health inequality in asthma burden would facilitate the evaluation of healthcare accessibility and health policy, identify obstacles of asthma diagnosis, treatment, and management, and propose potential strategies to mitigate these inequalities.

## Methods

### Data source

The GBD 2021 study conducted an extensive evaluation of 369 diseases, injuries, and impairments, alongside 87 risk factors, across 204 countries and territories [16, 17]. This evaluation was characterized by the utilization of the latest epidemiological data and refined standardized methodologies, ensuring the utmost precision and reliability of the findings. In this study, data on incidence, prevalence, deaths, and DALYs of asthma were extracted from the GBD 2021 (including 421 citations in the GBD 2021 Sources Tool), as well as asthma deaths, and DALYs attributable to smoking, high BMI, and occupational asthmagens (https://vizhub.healthdata.org/gbd-results/). Furthermore, the SDI was used as an economic indicator to rank countries and territories, as it reflected income, education, and fertility [18]. Information regarding the definition and calculation of the SDI was described in Appendix 1 of the supplementary methods.

### Asthma burden description

At the national level, the study examined the total asthma burden and asthma burden attributable to smoking, high BMI, and occupational asthmagens. Additionally, the population was categorized by age and sex. There were 5 age groups: <5 years, 5–14 years, 15–49 years, 50–69 years, and ≥ 70 years [16]. Crude rates of asthma incidence, prevalence, deaths, and DALYs were assessed for each age group. The GBD 2021 first uses CODEm and DisMod-MR to estimate age-, sex-, and region-specific disease indicators, then calculated age-standardized rates based on the 2019 age structure of the global population. The definition and estimation of asthma burden and attributable risk factors (smoking, high BMI, and occupational asthmagens) were presented in Appendix 2 of the supplementary methods. To avoid over-estimation of joint effects of risk factors, non-mediated relative risks were evaluated, and the above risks were assumed to have a multiplicative relationship. Measures of asthma burden in GBD 2021 were presented in multiple formats, including the numbers and age-standardized rates per 100,000 population.

### Statistical analyses

#### Decomposition analysis

The decomposition methodology of Das Gupta was used to isolate and quantify the additive contributions of individual risk factors to the complex outcome [19]. Through decomposition analysis, we elucidated contributions of distinct components to the change in asthma burden from 1990 to 2021, including three population-level determinants (age structure, population size, and epidemiologic change). By adopting this approach, we comprehensively evaluated the specific influence of each factor on the observed disparities across populations [19]. Further details were provided in Appendix 3 of the supplementary methods.

#### Cross-national health inequality

The study used two standard measures, the SII and RCI, according to the standard health equity analysis methodology recommended by the World Health Organization, to assess the inequalities in the cross-country distribution of asthma burden (including asthma burden attributable to risk factors) across all countries. To measure health inequality in the socioeconomic dimension across 204 countries and territories, countries and territories were ranked based on their SDI values, with ranks ranging from 0 (representing the most disadvantaged) to 1 (representing the most advantaged). To consider the proportional distribution of the population within each country, the assigned weighted rank corresponded to the midpoint of its cumulative proportional population. The SII, a vital component of the regression-based measure of social inequality, represents the slope of the regression line correlating the national asthma DALYs rate to the weighted ranking of countries (SDI increased from 0 to 1). It quantifies the absolute difference in predicted rates between the highest and lowest sociodemographic groups [20]. Additionally, RCI provides a comprehensive assessment of relative gradient inequality in asthma burden, and assesses the equitable allocation of resources. The RCI is determined by the relative location between the concentration curve and the diagonal line (also known as equal line). It ranges from −100 to100 and quantifies the cumulative proportion of the global asthma burden to the cumulative share of each country’s population, ranked by SDI. A negative RCI (concentration curve lines above equal line) indicated a concentration of asthma burden in countries with lower SDI values, while a positive RCI (concentration curve lines below equality line) suggested a concentration of asthma burden in countries with higher SDI values [20]. Methods of SII and RCI calculation were detailed in Appendix 4 of the supplementary methods. Comparisons of SII and RCI values between 1990 and 2021 were performed using a two-sample *t*-test, based on the uncertainty distributions of the estimates.

The rate of asthma burden was reported as an estimate per 100,000 population, along with its 95% uncertainty interval (*UI*, determined by the 2.5% and 97.5% of the 1,000 ordered estimates). The *UI* is used to reflect all the source of uncertainty and avoid over-interpretation of the point estimate. We calculated the upper and lower uncertainty limits for both the SII and RCI. All data analyses and visualizations were performed using the Health Equity Assessment Toolkit developed by the World Health Organization [18] (https://www.who.int/data/inequality-monitor) and R software (version 4.2.2, R core team, Vienna, Austria; the code for the health inequality analysis was shown in Appendix 5). A two-sample *t*-test was used for the comparisons of inequalities in the asthma incidence, prevalence, deaths, and DALYs rates between 1990 and 2021, and the two-sided *P* < 0.05 was defined as statistical significance.

## Results

### Burden and trends of asthma and attributable risk factors

**Fig. 1:**
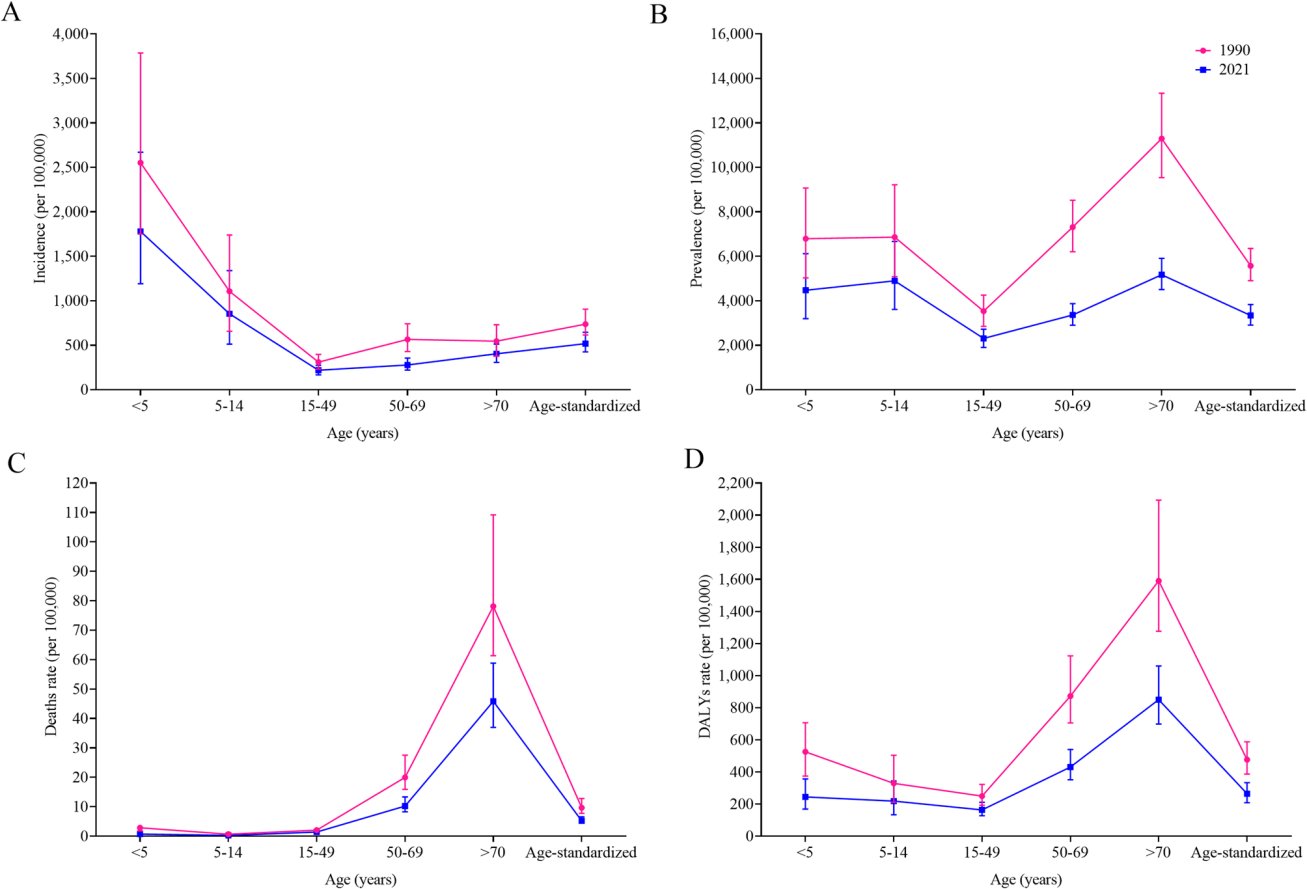
Age distribution of global asthma incidence, prevalence, deaths, and DALYs rates in 1990 and 2021. Notes: **A **Age distribution of global asthma incidence rate in 1990 and 2021; **B** Age distribution of global asthma prevalence rate in 1990 and 2021; **C** Age distribution of global asthma deaths rate in 1990 and 2021; **D** Age distribution of global asthma DALYs rate in 1990 and 2021

Global asthma rates of incidence, prevalence, deaths, and DALYs declined from 1990 to 2021, but they exhibited age-, sex-, and risk factor-specific distribution. Globally, the incidence rate, prevalence rate, deaths rate, and DALYs rate of asthma was 516.70 (95% *UI*: 425.36, 646.13), 3,340.12 (2,905.24, 3,832.24), 5.20 (4.27, 6.59), and 264.62 (208.32, 646.13) per 100,000 population in 2021, respectively; decreased by 29.89%, 40.01%, 46.08%, 44.46% during 1990–2021, respectively (Fig. 1). In both sexes, the age group under five years exhibited the highest asthma incidence, and asthma incidence first decreased and then increased after 50 years. People aged 70 years and over consistently demonstrated the highest prevalence, death, and DALYs rates of asthma, which declined across all ages and sexes (Figs. 1 and S1). Between 1990 and 2021, asthma deaths and DALYs rates associated with smoking, high BMI, and occupational asthmagens demonstrated a decline across all age groups in both sexes (Tables S1 and S2). Except for the highest asthma DALYs rate attributable to occupational asthmagens in the 50–69 age group, the highest burden of the remaining factors predominantly manifested among people aged ≥ 70 years (Table S2). Asthma burden attributable to smoking and occupational asthmagens was higher in the male than female, but asthma burden attributable to high BMI was higher in the female (Tables S1 and S2).

### Decomposition analysis of total and risk factor-specific asthma burden

**Fig. 2:**
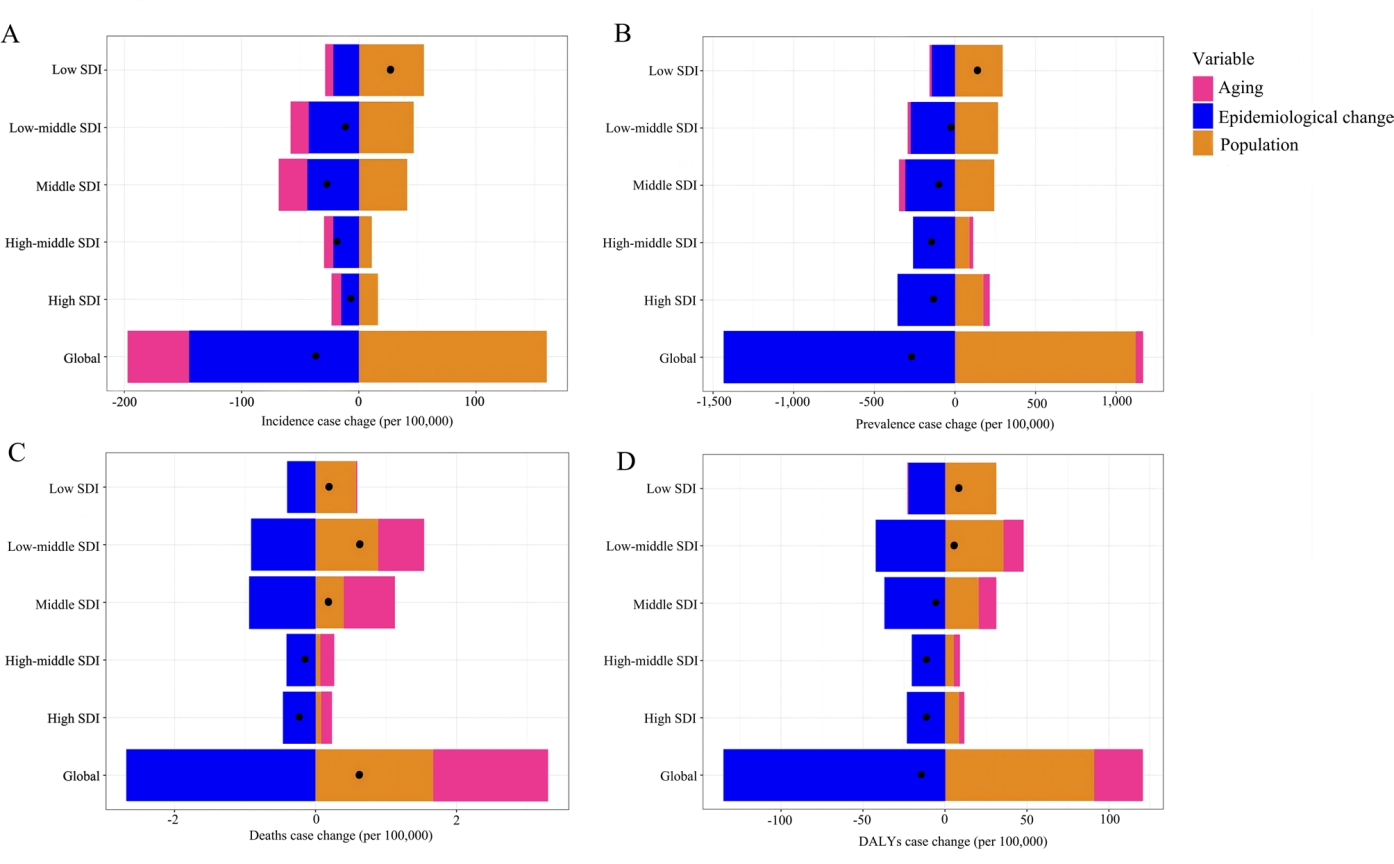
Decomposition analysis of global changes in incidence, prevalence, deaths, and DALYs case change of asthma according to population-level determinants during 1990–2021 by SDI quintile. Notes: **A** Decomposition analysis of incidence case change of asthma from 1990 to 2021 at the global level and by SDI quintile; **B** Decomposition analysis of prevalence case change of asthma from 1990 to 2021 at the global level and by SDI quintile; **C** Decomposition analysis of deaths case change of asthma from 1990 to 2021 at the global level and by SDI quintile; **D** Decomposition analysis of DALYs case change of asthma from 1990 to 2021 at the global level and by SDI quintile

Aging and population growth were leading contributors to increased asthma death, but epidemiological change contributed mostly to decreased numbers of asthma incidence, prevalence, and DALYs. Globally, the numbers of incident and prevalent asthma cases decreased, as well as asthma DALYs, which were mainly contributed by epidemiologic change (incidence: 391.75%, prevalence: 534.67%, and DALYs: 937.81%). Conversely, numbers of asthma incidence, prevalence, and DALYs cases increased in low SDI region, and population growth contributed 206.16%, 212.49%, and 374.16%, respectively (Fig. 2A, B and D, and Table S3). The impact of aging on asthma prevalent cases varied across different levels of SDI, leading to a decrease in middle, low-middle, and low SDI areas but an increase in other SDI areas (Fig. 2B). Aging and population growth contributed 127.48% and 138.99% to the increase in the global cases of asthma deaths, respectively; however, epidemiologic change contributed to the decrease in asthma deaths number in high and high-middle areas (Fig. 2C and Table S3). Results of the sex-specific decomposition analysis on the determinants of asthma burden were similar to those observed in the whole population (Figure S2).

The numbers of smoking-related asthma deaths and DALYs fell, while the numbers of high BMI-related asthma burden deaths and DALYs rose. Risk factor-specific decomposition analysis showed that epidemiological change led to decrease in the smoking-related asthma deaths and DALYs, especially in male (Table S4). However, the global cases of asthma deaths and DALYs attributed to high BMI increased significantly, which was driven primarily by aging and population growth, especially in low, low-middle, and middle SDI regions (Table S5). Occupational asthmagen-related asthma deaths increased slightly in the whole population and female (driven by aging and population growth), but decreased among the male (driven by epidemiological change); occupational asthmagen-related asthma DALYs decreased in the whole population and male (driven by epidemiological change), but raised in the female (Table S6).

### Cross-national health inequality in asthma burden, 1990 to 2021

**Fig. 3:**
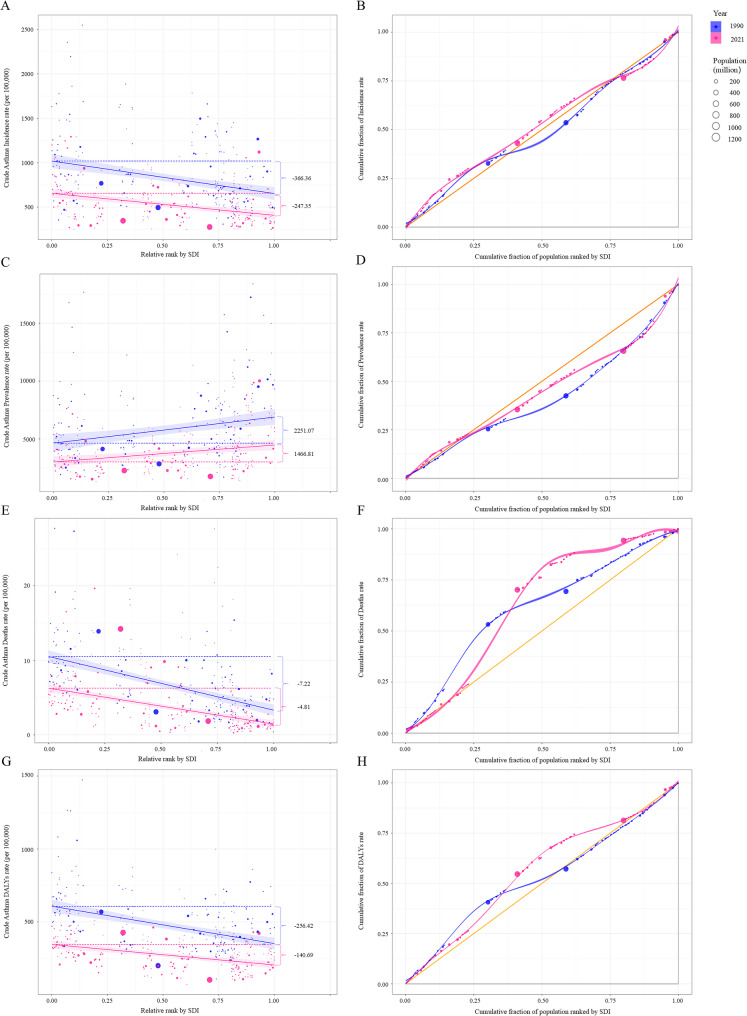
Health inequality regression curves and concentration curves for global asthma incidence, prevalence, deaths, and DALYs rates in 1990 and 2021. Abbreviations: RCI, relative concentration index; SII, slope inequality index. Notes: **A** SII of global asthma incidence in 1990 and 2021; **B** RCI of global asthma incidence in 1990 and 2021; **C** SII of global asthma prevalence in 1990 and 2021; **D** RCI of global asthma prevalence in 1990 and 2021; **E** SII of global asthma deaths rates in 1990 and 2021; **F** RCI of global asthma deaths rates in 1990 and 2021; **G** SII of global asthma DALYs rates in 1990 and 2021; **H** RCI of global asthma DALYs rates in 1990 and 2021. The SII (Panels **A**, **C**, **E**, **G**), derived from health inequality regression curves, represented the absolute inequality in global asthma incidence, prevalence, deaths, and DALYs rates across 204 countries and territories and the RCI (Panels **B**, **D**, **F**, **H**), derived from concentration curves, represents the relative inequality

Global inequalities in asthma incidence and prevalence narrowed, remained stable for asthma death, but widened in asthma DALYs. The SII of asthma incidence rate narrowed in the overall population, as the slope changed from −366.36 (95% *CI*: −505.84, −226.87) in 1990 to −247.35 (95% *CI*: −331.83, −162.88) in 2021, and countries with lower SDI had higher asthma incidence rate in both 1990 and 2021. The RCI of asthma incidence was close to 0 in 2021 [−0.27 (95% *CI*: −0.37, −0.16)], which indicated that countries with varying levels of SDI faced similar challenges of incident asthma. The gap in asthma prevalence rate between the highest and the lowest SDI countries decreased [the slope was 2,251.07 (95% *CI*: 1,115.31, 3,386.82) in 1990 vs. 1,466.81 (95% *CI*: 716.38, 2,217.24) in 2021]. The concentration curve for asthma prevalence lined below the diagonal line and the RCI improved from 23.92 (19.99, 27.85) in 1990 to 9.90 (8.64, 11.17) in 2021. The slope for asthma deaths and DALYs rates was negative, and the SII narrowed. In addition, there was no great improvement in the RCI of asthma deaths. But concentration curve for asthma DALYs rates lined above the diagonal line, and the RCI concentrated in countries with lower SDI and increased (Table 1; Fig. 3). The comparison of asthma-related health inequality between male and female revealed that the RCI was slightly higher in female than that in male (Table 1).

**Table 1:** Slope index of inequality and relative concentration index for age-standardized asthma incidence, prevalence, deaths, and DALYs rates, by sex, in 204 countries and territories, 1990–2021

Indices	SII (95% CI)			RCI (95% CI)		
	1990	2021	*P*	1990	2021	*P*
Both						
Incidence	−366.36 (−505.84, −226.87)	−247.35 (−331.83, −162.88)	< 0.001	−6.86 (−11.31, −2.41)	−0.27 (−0.37, −0.16)	< 0.001
Prevalence	2,251.07 (1,115.31, 3,386.82)	1,466.81 (716.38, 2,217.24)	< 0.001	23.92 (19.99, 27.85)	9.90 (8.64, 11.17)	< 0.001
Deaths rate	−7.22 (−8.58, −5.85)	−4.81 (−5.83, −3.79)	< 0.001	−26.09 (−41.72, −10.47)	−27.24 (−46.57, −7.90)	0.741
DALYs rate	−256.42 (−323.05, −189.80)	−140.69 (−182.26, −99.12)	< 0.001	−8.94 (−11.82, −6.05)	−12.28 (−16.77, −7.79)	< 0.001
Male						
Incidence	−406.65 (−550.05, −263.244)	−268.62 (−352.89, −184.36)	< 0.001	9.03 (6.67, 11.40)	1.80 (1.21, 3.45)	< 0.001
Prevalence	1,843.52 (707.03, 2,980.00)	1,126.42 (430.38, 1,822.46)	< 0.001	21.03 (17.16, 24.89)	10.80 (8.57, 13.02)	< 0.001
Deaths rate	−7.96 (−12.33, −8.72)	−4.91 (−5.95, −3.89)	< 0.001	−25.61 (−43.46, −7.77)	−20.46 (−38.92, −6.17)	0.130
DALYs rate	−305.05 (−376.86, −233.23)	−158.757 (−199.76, −117.76)	< 0.001	−9.89 (−13.58, −6.20)	−15.69 (−36.47, 5.08)	< 0.001
Female						
Incidence	−321.71 (−460.44, −182.97)	−220.72 (−307.32, −134.12)	< 0.001	11.84 (9.40, 14.28)	6.52 (5.89, 7.34)	< 0.001
Prevalence	2,645.96 (1,472.98, 3,818.94)	1,773.78 (955.47, 2,592.09)	< 0.001	26.57 (22.59, 30.54)	19.31 (18.83, 21.45)	< 0.001
Deaths rate	−6.30 (−7.59, −5.02)	−4.67 (−5.70, −3.65)	< 0.001	−26.59 (−47.57, −5.60)	−24.36 (−35.41, −5.77)	0.536
DALYs rate	−210.14 (−276.53, −143.76))	−124.10 (−168.80, −79.39)	< 0.001	−23.14 (−40.77, −5.51)	−18.59 (−37.19, 0.02)	< 0.001

Notes: *P* values represented comparisons of SDI-related inequalities in asthma burden between 1990 and 2021 using a two-sample *t*-test. The 95% *CI* was based on the robust regression coefficients and their standard errors, and was calculated using the normal distribution approximation. The SII derived from health inequality regression curves, represented the absolute inequality in asthma incidence, prevalence, deaths, and DALYs rates across 204 countries and territories and the RCI derived from concentration curves, represents the relative inequality

*Abbreviations: DALYs *disability-adjusted life years,* RCI *relative concentration index,* SII *slope inequality index, *CI* confidence interval

Smoking-related inequality in asthma deaths remained stable, high BMI-related inequality in asthma deaths worsened, but inequalities in risk factor-specific asthma DALYs all narrowed. As illustrated by SII, the gap in asthma death rate attributable to smoking was nearly unchanged in the general population. The RCI showed that smoking attributable asthma death rate was concentrated in countries with lower SDI (Table 2 and Figures S3A-S3B). SII of smoking-related asthma DALYs rate was 20.30 (95% CI: 16.26, 40.75) in 1990 and 6.87 (95% *CI*: 2.96, 10.78) in 2021. What’s more, RCI of smoking-related asthma DALYs rates were concentrated in countries with higher SDI, but this pattern improved from 1990 to 2021 (Table 3 and Figures S3C-S3D). The SII of high BMI-related asthma deaths rates decreased from −0.10 (95% *CI*: −0.43, 0.16) in 1990 to −0.53 (95% *CI*: −0.75, −0.31) in 2021, and the RCI of high BMI-related asthma deaths rates changed from 0.80 (95% *CI*: −0.25, 1.84) in 1990 to −18.78 (95% *CI*: −36.33, −1.22) in 2021 (Table 2 and Figures S4A-S4B). Moreover, the RCI of asthma DALYs rate due to high BMI was 31.50 (95% *CI*: 21.14, 45.72) in 1990 and 12.83 (95% *CI*: 2.95, 22.70) in 2021, and the RCI of asthma DALYs rate attributable to high BMI had a similar trend with SII in the whole population (Table 3 and Figure S4). In the general population, SII of occupational asthmagens-related asthma deaths rates changed from −0.91 (95% *CI*: −0.65, −1.72) in 1990 to −0.64 (95% CI: −0.73, −0.55) in 2021, but the RCI of occupational asthmagens-related asthma deaths remained stable (Table 2 and Figures S5A-S5B). Both SII and RCI of occupational asthmagens-related asthma DALYs were negative in 2021 and narrowed in the whole population (Table 3 and Figure S5). When comparing health inequalities in asthma deaths and DALYs rate attributable to three risk factors between male and female, RCI of smoking- and occupational asthmagens-related asthma death was slightly higher in male than female, but RCI of high BMI-related asthma deaths was slightly higher in female. RCI of asthma DALYs were also slightly higher in female except for asthma DALYs attributable to occupational asthmagens (Table 3).

**Table 2:** Slope index of inequality and relative concentration index for asthma deaths rates attributed to smoking, high BMI and occupational asthmagens, by sex, in 204 countries and territories, 1990–2021

Risk factors	SII (95% CI)			RCI (95% CI)		
	1990	2021	*P*	1990	2021	*P*
Both						
Smoking	−0.14 (−0.56, 0.02)	−0.19 (−0.27, −0.11)	0.059	−15.61 (−26.92, −4.30)	−22.09 (−64.70, 20.52)	0.295
High BMI	−0.10 (−0.43, 0.16)	−0.53 (−0.75, −0.31)	< 0.001	0.80 (−0.25, 1.84)	−18.78 (−36.33, −1.22)	< 0.001
Occupational asthmagens	−0.91 (−0.65, −1.72)	−0.64 (−0.73, −0.55)	< 0.001	−45.02 (−102.45, 12.41)	−34.21 (−66.49, −1.94)	0.242
Male						
Smoking	−0.38 (−0.64, 0.12)	−0.39 (−0.52, −0.26)	0.859	−18.40 (−32.77, −4.04)	−20.90 (−40.31, −3.50)	0.445
High BMI	0.07 (−0.16, 0.29)	−0.46 (−0.66, −0.26)	< 0.001	1.95 (−0.23, 4.12)	−14.38 (−29.21, −1.63)	< 0.001
Occupational asthmagens	−1.15 (−1.32, −0.99)	−0.75 (−0.85, −0.64)	< 0.001	−36.18 (−67.01, −5.36)	−30.21 (−58.33, −7.69)	0.286
Female						
Smoking	0.09 (0.02, 0.15)	−0.01 (−0.04, 0.03)	< 0.001	0.74 (−0.37, 1.85)	−18.76 (−32.54, 2.01)	< 0.001
High BMI	−0.26 (−0.52, 0.01)	−0.59 (−0.84, −0.34)	< 0.001	−0.31 (−0.62, 0.01)	−16.23 (−33.24, 2.78)	< 0.001
Occupational asthmagens	−0.62 (−0.72, −0.52)	−0.52 (−0.60, −0.45)	< 0.001	−38.49 (−71.20, −5.78)	−24.63 (−63.57, −12.79)	0.017

Notes: *P* values represented comparisons of SDI-related inequalities in asthma death rate attributed to risk factors between 1990 and 2021 using a two-sample *t*-test. The 95% *CI* was based on the robust regression coefficients and their standard errors, and was calculated using the normal distribution approximation. The SII derived from health inequality regression curves, represented the absolute inequality in asthma deaths rates attributed to smoking, high BMI and occupational asthmagens across 204 countries and territories and the RCI derived from concentration curves, represents the relative inequality

*Abbreviations: BMI* body mass index, *RCI* relative concentration index, *SII* slope inequality index, *CI* confidence interval

**Table 3:** Relative concentration index and slope index of inequality for asthma DALYs rate attributed to smoking, high BMI and occupational asthmagens, by sex, in 204 countries and territories, 1990–2021

Risk factors	SII (95% CI)			RCI (95% CI)		
	1990	2021	*P*	1990	2021	*P*
Both						
Smoking	20.30 (16.26, 40.75)	6.87 (2.96, 10.78)	< 0.001	11.82 (5.51, 18.14)	−0.61 (−1.31, 0.08)	< 0.001
High BMI	31.50 (21.14, 45.72)	12.83 (2.95, 22.70)	< 0.001	22.77 (9.89, 35.64)	4.87 (1.96, 7.78)	< 0.001
Occupational asthmagens	−14.57 (−12.65, −15.15)	−15.98 (−20.78, −11.18)	< 0.001	−33.78 (−64.53, −3.02)	−13.02 (−19.67, −6.38)	< 0.001
Male						
Smoking	16.10 (5.56, 26.64)	0.53 (−4.76, 5.81)	< 0.001	1.40 (0.29, 2.51)	−5.85 (−9.89, −2.36)	< 0.001
High BMI	34.16 (23.61, 44.71)	35.31 (25.42, 45.19)	0.570	22.06 (7.67, 36.45)	5.62 (2.44, 12.92)	< 0.001
Occupational asthmagens	−18.01 (−26.96, −9.06)	−18.77 (−24.16, −13.39)	0.604	−13.99 (−21.28, −6.71)	−13.71 (−21.65, −7.75)	0.843
Female						
Smoking	23.10 (17.61, 28.59)	12.84 (9.47, 16.20)	< 0.001	40.62 (17.05, 64.18)	29.48 (11.97, 46.04)	0.006
High BMI	27.05 (14.94, 39.16)	13.63 (1.83, 25.43)	< 0.001	23.03 (9.51, 36.54)	8.46 (3.10, 13.83)	< 0.001
Occupational asthmagens	−11.84 (−18.05, −5.63)	−12.47 (−17.03,−7.91)	0.560	−7.25 (−10.50, −4.00)	−3.88 (−5.32, −2.68)	< 0.001

Notes: *P* values represented comparisons of SDI-related inequalities in asthma DALYs rate attributed to risk factors between 1990 and 2021 using a two-sample *t*-test. The 95% *CI* was based on the robust regression coefficients and their standard errors, and was calculated using the normal distribution approximation. The SII derived from health inequality regression curves, represented the absolute inequality in asthma DALYs rates attributed to smoking, high BMI and occupational asthmagens across 204 countries and territories and the RCI derived from concentration curves, represents the relative inequality

*Abbreviations: BMI* body mass index, *RCI* relative concentration index, *SII* slope inequality index

## Discussion

This study quantified trends, contributors, and health inequality in asthma burden during 1990–2021. There was a dramatic decrease in asthma burden in both sexes and all age groups, as well as risk factors-related asthma burden. Decomposition analysis showed that epidemiological change was the leading driver of the decreased number of asthma incidence, prevalence, and DALYs, aging and population growth contributed to the increased number of asthma deaths. Health inequality in asthma incidence and prevalence narrowed during 1990–2021, as well as risk factors-related asthma DALYs, but inequality in smoking- and high BMI-related asthma deaths widened and were more obvious in countries with lower SDI. Our findings highlighted that health system reforms should prioritize disadvantaged subgroup of populations and health inequality in high-BMI and occupational asthmagens related asthma death, and reinforce obesity intervention, occupational protection, and asthma management in the region with low SDI. The implementation of tobacco control policies should be continuously pushing forward, including higher tobacco tax and public smoking bans.

Trend analysis showed that the incidence rate of asthma was the highest in children aged 0–5 years and showed a “V”-shaped trend; however, the prevalence, death, and DALYs rates of asthma in the population aged ≥ 70 years were at the highest level. Some factors can explain those results. It is widely accepted that asthma originates in childhood [21], and children had the highest asthma incidence due to their vulnerability to respiratory virus infections and other environmental hazards [22]. Incidence of allergic asthma decreased with increasing age firstly, it was low in childhood and early adulthood, but then increased and peaked in 50–59 years old [23]. Boys have higher childhood asthma prevalence, while adult women higher prevalence and severity, driven by sex hormones, genetic variants/epigenetic modification, social/environmental factors, and asthma therapeutic response [24]. The chronic nature of the asthma and cumulative exposure to risk factors may contribute to a higher asthma prevalence, deaths, and DALYs in older adults. Age-related changes in lung function can increase the risk of developing or exacerbating existing asthma symptoms, and common comorbidities in older adults, such as COPD, cardiovascular disease, and obesity, could interact with asthma and lead to misclassification with COPD/overdiagnosis of asthma and worsen symptoms [25, 26]. The severity of small airway disease was more pronounced in older people compared to their younger counterparts [27], and the prognosis for late-onset asthma is comparatively unfavorable [28]. Consequently, older adults had the highest asthma deaths and DALYs rates, and the global asthma deaths and DALYs rates attributable to smoking, high BMI, and occupational asthmagens were highest in the population aged ≥ 70 years. Smoking- and occupational asthmagens-related asthma deaths rate was higher in the male than that in female, which may be due to higher prevalence of smoking and risk of exposure to occupational asthmagens in male. Addressing the heterogeneity of asthma burden across diverse age groups is crucial for proactively managing this chronic condition, thereby alleviating asthma burden and enhancing their quality of life.

Epidemiologic change has a dramatic influence on the decrease in asthma incidence, prevalence, and DALYs number, and demographic expansion (population growth and aging) contributed to an increase in the asthma deaths number. Because the number of incident asthma was mostly observed in children, population growth alleviated the reducing trend in the number of asthma incidence except in the low SDI regions. The effect of aging on the asthma deaths and DALYs was greater in areas with higher SDI, while population growth emerged as a strong determinant in lower SDI areas, which were predominantly characterized by high fertility rates and low life expectancy [29]. Aging is associated with diverse health implications in different nations and regions. International organizations and national governments should consider these factors when addressing the potential health impacts of aging, and develop tailored solutions to meet local needs. Our analysis indicated that asthma DALYs number due to smoking and occupational asthmagens decreased because of epidemiological changes. The global prevalence of daily smoking has experienced a significant decline of 29.4% since 1990 [30], meanwhile the global burden of asthma attributable to occupational risks also exhibited a notable reduction [31]. Then, both aging and population growth contributed to increase in high BMI-related asthma deaths and DALYs, especially in middle and low-middle SDI regions. Decomposition analysis helps to reveal the driver of asthma burden in different SDI regions.

Inequalities in incidence, deaths, and DALYs rates of asthma concentrated in countries with lower SDI, but inequalities of asthma prevalence concentrated in countries with higher SDI, and they showed different trends over time. Increased exposure to indoor allergens and pollutants, abuse of antibiotics, westernized diets and lifestyle, diagnostic biases of asthma (better detection in high-SDI regions) and improved survival may contribute to high asthma prevalence in high SDI countries and discrepancies in asthma prevalence. There was also limited data resources of asthma prevalence in the lower SDI regions, and the GBD study can’t fully account for these diagnostic disparities due to insufficient data availability. Despite lower documented prevalence, low SDI countries exhibit higher asthma deaths and DALYs rates, likely attributable to underdiagnosis and survival differences. High-income countries with advanced healthcare systems demonstrate effective asthma management, yielding low asthma deaths and DALYs rates [32]. In low- and middle-income countries, widespread use of biomass, ambient air pollution, underdiagnosis and limited availability of inhaled corticosteroids, biologics, and emergency care—due to challenges in treatment affordability, healthcare accessibility, and effectiveness—contributes to increased asthma incidence, suboptimal asthma control and increased risk of fatal exacerbations. Low-income countries have developed locally tailored measures to optimize existing primary healthcare services [33], which would reduce inequality in asthma deaths and DALYs. Noteworthy, the magnitude of absolute difference in asthma deaths between countries with extreme SDIs decreased, but relative inequality in asthma deaths persisted despite the overall improvement.

Besides, absolute and relative inequalities exhibited diverging trends in asthma attributable to specific risk factor. Increase in asthma death rates due to smoking and high BMI were both observed in low SDI areas, while asthma DALYs rate due to high BMI tended to concentrate in countries with higher SDI. Tobacco use prevalence was high in low SDI countries, especially the South-East Asia Region [34], and high smoking prevalence put them at increased risk of falling into severe asthma symptoms and steroid insensitivity, which may lead to high death rates. While tobacco consumption declined in high- and high-middle income countries, it exhibited a significant increase in developing regions and most smokers resided in the lower SDI regions [35]. Similarly, with few exceptions, the smoking rates in most low- and middle-income SDI countries barely declined [36]. The high asthma burden due to smoking in low SDI areas may also be related to biological and genetic factors. For instance, the Europeans exhibited significantly lower methylation levels at the *AHRR* site (A smoking-related DNA methylation locus, and it has been confirmed to be associated with potential impairment of lung function and increased susceptibility to respiratory diseases [37]) compared to South Asians [38]. potentially reflecting the effective implementation of more comprehensive tobacco control policies [39]. High BMI became the top risk factor for asthma-related deaths in the past three decades according to the GBD 2021 study [40]. There was nearly no inequality in asthma death associated with high BMI in 1990, but inequality widened and concentrated in lower SDI regions in 2021. Obesity can lead to changes in respiratory mechanics, including increased respiratory effort and reduced strength of the respiratory muscles, which would worsen respiratory symptoms and asthma control, and increased the risk of asthma death [41, 42]. Increasing prevalence of obesity, inadequate and poor-quality medical care led to a higher burden of high BMI-related asthma death in lower SDI regions. Socio-economic determinants of obesity varied in different SDI regions. Due to cheap and easily accessible of high-fat and high-sugar food, the obesity epidemic has reached a more intricate stage and maintained at a high prevalence in developed regions [12]. In the middle SDI regions, countries are facing rapid urbanization, transition to the westernized diets and sedentary lifestyle, and enlarged urban-rural difference. The surge in obesity rates has precipitated the paradoxical emergence of “double malnutrition” (under- and over-nutrition) in the low SDI regions, obesity has increasingly become an indicator of poverty, owing to westernized diets [43], calorie-micronutrient imbalance, and limited healthcare for obesity management. Besides, the prevalence of obesity-related comorbidities, including hypertension and diabetes, was higher [44], and the occurrence of these comorbidities may further exacerbate asthma burden [45]. These reasons may explain the increasing concentration of asthma burden attributable to high BMI in low SDI region. With the simultaneous increase in population aging and life expectancy within these countries [46], higher rates of asthma DALYs attributable to high BMI continued to be concentrated in areas with higher SDI. Sex disparities may exist in the impact of obesity on asthma DALYs, which was more pronounced in female [47]. Based on GBD data, age-standardized global rate of high BMI was slightly higher in the female than that in the male. There was sex-specific variation in the obesity-asthma association, and endogenous sex hormones may serve as the potential effect modifiers. Pubertal hormonal fluctuation acts as a critical biological determinant influencing asthma burden in the girls, leading to increased asthma burden from childhood to puberty and adulthood [48]. Obesity-influenced estrogen would enhance Th2 response, and increase the production of IL-4 and IL-13; estrogen also reduces response to inhaled corticosteroid. However, testosterone decreases innate immune response. Furthermore, exogenous estrogen administration through hormone replacement therapy has been epidemiologically linked to elevated asthma risk in postmenopausal female [49]. Moreover, another study found that sex differences in asthma burden were most pronounced in low SDI areas and narrowed in middle SDI areas [5]. This could partly explain why inequality in asthma burden due to high BMI was higher in female than that in male in the low SDI regions. While this study provides a comprehensive overview of the population-level burden of asthma, it is important to note that the GBD estimates are not stratified by treatment setting. The total prevalent and incident asthma cases encompass those managed in primary care, those requiring emergency department visits, and those severe enough to necessitate hospitalization. This aggregation means our estimates reflect the overall community burden but do not directly quantify the specific burden within different tiers of the healthcare system. For instance, a high asthma prevalence rate might predominantly reflect well-controlled cases in primary care, while the high deaths or DALYs rate likely signals a significant burden on inpatient and specialist services. Asthma and obesity are often treated as separate disorders in primary care, and the link between them is often overlooked. However, reducing BMI through dietary changes and physical activity is essential for effective asthma control. Specialized outpatient clinics pay more attention to pharmacologic treatment. A more holistic, multidisciplinary approach involving pulmonologists, dietitians, and physiotherapists could improve patient outcomes. High BMI is also associated with more frequent asthma exacerbations in hospital and emergency settings, leading to higher hospitalization rates and healthcare costs. Therefore, weight management should be an integral part of long-term asthma care to reduce exacerbations and healthcare burden. The policymakers in the lower SDI regions should reinforce obesity management and intervention, including providing healthy foods, improving health infrastructure, and enhancing obesity treatment. Asthma should be treated as a heterogeneous disease and the implementation of tobacco control policies should be continuously advanced. In primary care, smoking cessation counseling was often overlooked in routine asthma care, whereas in emergency and inpatient settings, acute exposure often triggered severe attacks requiring intensive treatment. Systematically integrating smoking screening and cessation support across all healthcare settings was essential to reduce acute asthma episodes and improve long-term disease control.

Asthma deaths and DALYs rates attributable to occupational asthmagens concentrated in low SDI regions and tended to narrow. Occupational asthma is the second most prevalent lung disease in developing regions, such as Africa and South Asia [14]. The latest report by the GBD 2021 Chronic Respiratory Diseases Collaborators revealed that South and East Asia exhibited the highest DALYs rate for chronic respiratory diseases attributable to occupational hazards [5]. The occupational protection measures that can significantly reduce the burden of occupational asthma were established earlier in developed countries [50]. The regulation and enforcement of occupational health and safety standards were often less stringent in developing countries, leading to increased exposure to hazardous substances [51]. Moreover, limited knowledge regarding emerging occupational asthmaengs exposure contributed to the underestimation of the burden of work-related asthma [52]. A variety of loose occupational exposure limits have been proposed in Asian countries [53]. The implementation of multiple occupational protection measures by developing nations and enhancing awareness of occupational safety and health protection may partially account for the recent improvement in health inequities in occupational asthmagens-related asthma burden. Occupational asthmagens present unique challenges due to their workplace-specific environmental persistence. In primary care, providers focus on obtaining occupational histories to identify asthma triggers. In contrast, in specialist or inpatient settings, removing patients from exposure was critical—alongside targeted pharmacotherapy—to prevent recurrent exacerbations and long-term disability.

The strengths of this study are listed as follows. It is the first study to decompose the asthma burden attributable to demographic factors and epidemiological change, as well as stratified by sex. Furthermore, this study comprehensively assesses socioeconomic inequalities in the total and risk factor-specific asthma burden, thereby it contributes valuable evidence for future health policies aiming at addressing asthma disparities. The current study has several limitations. Firstly, the inherent limitations of GBD studies have been previously described [5]. Asthma diagnosis was based on self-reported wheezing and physician diagnoses, but recall bias, limited healthcare access, and differences in the interpretation of asthma questionnaire may affect estimates of asthma burden. Our reported asthma burden estimates may diverge from findings in controlled clinical datasets and registry-based studies. Differences may arise from case definitions (symptom-based, ICD-coded, or self-report), underdiagnosis in low-resource settings, overdiagnosis or asthma-COPD overlap among older adults, model-based adjustments and priors used in GBD, and temporal changes in diagnostic criteria and coding practices. Accuracy of estimates of asthma burden was compromised in regions with low and low-middle SDI, potentially leading to misdiagnosis or missed diagnosis due to inadequate asthma case registration and limited access to local medical resources, enlargement of inequality in asthma prevalence, and narrowness of inequality in asthma death, despite the use of rigorous statistical methods. Socioeconomic barriers in impoverished African regions, including illiteracy, limited healthcare access, and financial constraints, likely prevent accurate reporting of asthma burden [54]. The annual updates of GBD estimates could address these limitations by refining both the data collection system (especially in low SDI regions) and GBD methodology. These challenges also suggest the risk of model borrowing, where data gaps in one country are filled with estimates from regions with better reporting, which may not always reflect local realities. Secondly, the adjustment for confounding variables such as smoking, COPD overlap, and allergic conditions remains limited in GBD’s current methodology. These comorbidities and phenotypes can influence both asthma burden estimates and risk attribution, potentially distorting the observed relationship between asthma and its risk factors. Thirdly, another key limitation is the inability to infer causality from the ecological, model-based associations used in GBD. The data reflects associations, not causal relationships, which restricts our ability to make definitive conclusions about the direction of associations between risk factors and asthma burden. Furthermore, the absence of detailed treatment setting information, disease severity, or control status—such as exacerbation frequency, medication step, and clinical outcomes—limits the clinical relevance and interpretation of the findings. Fourthly, the decomposition analysis in our study focuses solely on the major demographic factors. Further studies could perform decomposition analysis of other environmental risk factors, quantify their contribution to the change in the number of multiple asthma measures, and identify the primary risk factor. Fifthly, our analysis is based on country-level data of asthma burden, thus failing to capture intra-country inequalities, which may be more important [55]. Country-level data masked important internal variations. The GBD study does not incorporate advanced stratification, including urban/rural residency, domestic regional differences, occupational exposure, and asthma phenotypes, and was unable to fully integrate multimorbidity. This underscores the importance of more nuanced stratification in future research to better account for these factors. GBD collaborators should collect and release state or even county level data of disease burden, like the United Kingdom and the United States of America. Sixthly, SII and RCI are univariate indicators of inequality in the present study, thus lacking information on the correlation between health inequality [56] and other social indicators [57]. Further study could replace SDI with other social indicators when explore asthma inequality, like educational attainment and household wealth. Lastly, the observed wide *UI* may be due to limited number of available data resources, studies with small sample sizes, or even conflicting data. Moreover, it is important to note that diagnostic and coding changes over time could influence observed trends in asthma prevalence and deaths, independent of true shifts in the burden.

## Conclusion

This study clarified drivers of change in asthma burden and quantified total and risk factor-specific inequality. The leading drivers of numbers of asthma incidence, prevalence, deaths, and DALYs were not exactly the same (aging and population growth for deaths but epidemiological change for other indices). Inequality in asthma incidence, deaths, and DALYs concentrated in lower SDI regions, but asthma prevalence narrowed and concentrated in higher SDI regions. Although high BMI-related asthma death decreased, but inequalities increased and concentrated in lower SDI regions. Further study should focus on inequality in the high-BMI and occupational asthmagens related asthma death, and strengthen obesity intervention, occupational protection, and asthma management in the low SDI regions.

## Supplementary Information

Supplementary Material 1.

## Data Availability

The datasets generated and/or analyzed during the current study are publicly available from the Global Burden of Disease Study 2021 results tool, hosted by the Institute for Health Metrics and Evaluation (IHME). Persistent URL: https://vizhub.healthdata.org/gbd-results/. The code used for data query, aggregation, and analysis in this study is available from the corresponding author upon reasonable request.
