# Supplementary material for: Cross-country health inequality in the asthma burden: findings from the global burden of disease study 2021
Source: BMC Public Health. 2025 Nov 17;25:3972. doi: 10.1186/s12889-025-25149-y (PMC12625390; doi:10.1186/s12889-025-25149-y)
Supplement: Supplementary file 1 — Supplementary Material 1. [file 12889_2025_25149_MOESM1_ESM.pdf]

Supplemental Materials

Cross-country health inequality in the asthma burden: Findings from the Global Burden of Disease Study 2021

Siying Zhang[1,2,3,6,#], Yumei Zhong[1,2,3,#], Yingjie Teng[4], Lijun Tang[1], Yun Zhou[1], Wenge Li[1], Zongshi Gao[4], Hui Gao[5], Fang-biao Tao[1,2,3], Xiulong Wu[1,2,3]

Affiliations:

[1]Department of Maternal, Child and Adolescent Health, School of Public Health, Anhui Medical University, No 81 Meishan Road, Hefei 230032, Anhui, China

[2]Key Laboratory of Population Health Across Life Cycle (Anhui Medical University), Ministry of Education of the People's Republic of China, No 81 Meishan Road, Hefei 230032, Anhui, China

[3]Anhui Provincial Key Laboratory of Environment and Population Health across the Life Course, Anhui Medical University, No 81 Meishan Road, Hefei 230032, Anhui, China

[4]The First Clinical college of Anhui medical university, Anhui Medical University, No 81 Meishan Road, Hefei 230032, Anhui, China

[5]Department of Pediatrics, The First Affiliated Hospital of Anhui Medical University, No 218 Jixi Road, Hefei, 230022, Anhui, China

[6]Wujing Community Healthcare Service Center of Minhang District, Shanghai, 201100, China.

[#]These authors contributed equally to this work.

Contents

Appendix 1 Calculation of Socio-demographic Index.

Appendix 2 The definition and estimation of asthma burden and the risk factors.

Appendix 3 Decomposition analysis.

Appendix 4 Methods of slope index of inequality and relative concentration index calculation.

Appendix 5 Code for health inequality analysis.

Table S1 Age-specific global asthma deaths rate attributed to risk factors in 1990 and 2021.

Table S2 Age distribution of global asthma DALYs rate attributed to risk factors in 1990 and 2021.

Table S3 Changes in asthma burden according to population-level determinants and causes from 1990 to 2021.

Table S4 Changes in asthma burden attribute to smoking according to population-level determinants of population growth, aging, and epidemiological change from 1990 to 2021.

Table S5 Changes in asthma burden attribute to high BMI according to population-level determinants of population growth, aging, and epidemiological change from 1990 to 2021.

Table S6 Changes in asthma burden attribute to occupational asthmagen according to population-level determinants of population growth, aging, and epidemiological change from 1990 to 2021.

Figure S1 Age distribution of global asthma incidence, prevalence, deaths, and DALYs rates in 1990 and 2021, by sex.

Figure S2 Decomposition analysis of global changes in incidence, prevalence, deaths,

and DALYs case change of asthma according to population-level determinants during 1990-2021, by SDI quintile and sex.

Figure S3 Health inequality regression curves and concentration curves for asthma deaths and DALYs rates attributed to smoking, 1990 and 2021.

Figure S4 Health inequality regression curves and concentration curves for asthma deaths and DALYs rates attributed to high BMI, 1990 and 2021.

Figure S5 Health inequality regression curves and concentration curves for asthma deaths and DALYs rates attributed to occupational asthmagens, 1990 and 2021.

Appendix 1: Calculation of Socio-demographic Index

Previous study has provided a comprehensive description of the calculation methods for Social Development Index (SDI) and SDI quintiles[1]. The SDI was a comprehensive indicator combining three key indices: lag-distributed income per capita, average education level among individuals ≥15-year-old, and total fertility rate among individuals <25 year-old. Each index was scaled from 0 to 1. To evaluate the composite SDI for a particular location and year, the geometric mean of these three indices is calculated. That provided an overall assessment of the social development. For ease of interpretation and reporting, SDI value was multiplied by 100, resulting in a standardized scale ranging from 0 to 1. In the GBD 2021 study, SDI quintiles were established based on the computed SDI cutoffs using country-level estimates. Countries with populations less than 1 million were excluded from the calculations. Consequently, all locations were assigned to the appropriate quintiles based on their SDI value in the year 2021, facilitating comparative analysis within and across regions.

Appendix 2: The definition and estimation of asthma burden and the risk factors

Definition and estimation of asthma burden

In the study, asthma was defined as the presence of both a doctor's diagnosis and wheezing within the previous year. This definition adheres to the criteria outlined in the International Statistical Classification of Diseases and Related Health Problems, specifically categorized as J45-J46 in ICD-10 and 493 in ICD-9. Four alternative definitions were applied: self-reported asthma within the past year, self-reported lifetime asthma, only a doctor's diagnosis within the past year, and only wheezing within the past year. By incorporating these diverse definitions, our study enables a thorough evaluation of asthma incidence and prevalence. The main modeling tool utilized for asthma was DisMod-MR 2.1, and it allowed for non-linear relationship using cubic splines. The maximum remission parameter was set at 0.3, representing the upper bound of the largest observed data. Additionally, no data were available for the 0-0.5 age group due to the challenge of asthma diagnosis in very young infants. DisMod model incorporated several covariates to enhance the comprehensiveness and robustness of the estimation. These covariates included log lag distributed income, standardized exposure variables for asthma, healthcare services, and a quality index. By incorporating these variables,

the model aimed to improve the accuracy of the estimates[2]. In order to facilitate direct comparisons between various case definitions and/or study designs, MR-BRT models other than DisMod were used to improve the bias adjustment. To estimate asthma burden, GBD 2021 study employed the CODEm approach. Death models were developed for both sexes and various age groups, ranging from 1 year old to over 95 years old. Disability-Adjusted Life Years (DALYs) served as the metric for assessing asthma burden. DALYs are calculated as the sum of years lived with disability and years of life lost due to premature death. To estimate the proportion of individuals with different asthma severity levels, data from the USA Medical Expenditure Panel Surveys during 2000-2014 were utilized. Estimated proportions were 36.2% asymptomatic, 19.9% controlled, 20.6% partially controlled, and 23.3% uncontrolled asthma. Disability weights of 0, 0.015, 0.036, and 0.133 were assigned to above severity categories, respectively[2]. By considering the population size and asthma prevalence in different SDI subgroups, the total number of asthma cases and severity-specific cases were calculated. Severity-specific disability weights were then applied to estimate the number of years lived with disability, accounting for comorbidities. Sex-specific death rates were estimated for different age groups using the CODEm approach. These rates were then multiplied by the population size in each age group to obtain the number of deaths. In the GBD 2021, standard life tables were employed to compute YLL for each subgroup by applying age-specific mortality rates to estimate the potential loss of life expectancy due to premature mortality. This approach uses the expected remaining lifespan at each age to quantify the years of life lost relative to a standard population.

Definitions of asthma-related risk factors

High BMI for adults (older than 20 years) was defined as a BMI greater than 25.0 kg/m^2 and obesity was defined as BMI ≥30.0 kg/m^2. For children aged 1-19 years, high BMI was defined according to International Obesity Task Force standards[2]. Smokers including current smokers and former smokers. Current smokers were the individuals who occasionally or daily smoked, and former smokers were these who have quitted tobacco products for at least six months. Data for occupational risks were from the International Labor Organization, in which 22

occupational asthmagens were recorded[2]. Proportion of occupational exposure to asthmagens was estimated according to the population distribution of nine occupational groups[2].

Appendix 3: Decomposition analysis[3]

In our study, we applied the decomposition methodology of Das Gupta provided by Yan Xie to analyze asthma burden based on GBD data[3]. This methodology provided a framework for assessing the individual impacts of three crucial factors: population age structure (such as shifts in the distribution of different age groups), population growth (changes in the total population size), and epidemiologic change (alterations in the prevalence or incidence of asthma). The number of the incidence, prevalence, deaths, and DALYs of asthma were obtained from the following formula, as well as asthma deaths and DALYs attributed to risks factors at each location:

$$\text{i) Incidence}_{\,ay,\,py,\,ey} = \sum_{i=1}^{20}(a_{i,y} * p_y * e_{i,y})$$

$$\text{ii) Prevalence}_{\,ay,\,py,\,ey} = \sum_{i=1}^{20} a_{i,y} * p_y * e_{i,y})$$

$$\text{iii) Deaths}_{\,ay,\,py,\,ey} = \sum_{i=1}^{20}(a_{i,y} * p_y * e_{i,y})$$

$$\text{iv) DALYs}_{\,ay,\,py,\,ey} = \sum_{i=1}^{20}(a_{i,y} * p_y * e_{i,y})$$

Asthma-burden (incidence, prevalence, deaths, and DALYs) $_{ay,\ py,\ ey}$ meant DALYs case number based on the factors of age structure, population growth, and the rate of asthma burden in specific year y. The $a_{i,y}$ was the proportion of population for the age category *i* of the age categories in specific year y (the incidence, prevalence, deaths, and DALYs of asthma, and the deaths and DALYs of asthma attributed to high BMI were divided into 20 age categories; the deaths and DALYs of asthma attributed to smoking was divided into 14 age categories; and the DALYs and deaths of asthma attributed to occupational asthmagens was divided into 17 age categories); p_y showed the general population in the given year y; and $e_{i,y}$ represented the rate of asthma burden of the age category *i* in year y. In our analysis of asthma burden from 1990 to 2021, the individual impact of each factor was determined by isolating the effect of a

single factor while keeping the remaining factors unchanged. As an example, the effect of age structure of incidence was calculated as:

$$[(\text{Incidence}_{a2021,\,p1990,\,e1990} + \text{Incidence}_{a2021,\,p2021,\,e2021})/3 + (\text{Incidence}_{a2021,\,p1990,\,e2021} + \text{Incidence}_{a2021,\,p2021,\,e1990})/6] - [(\text{Incidence}_{a1990,\,p2021,\,e2021} + \text{Incidence}_{a1990,\,p1990,\,e1990})/3 + (\text{Incidence}_{a1990,\,p2021,\,e1990} + \text{Incidence}_{a1990,\,p1990,\,e2021})/6]$$

The effect of population changes and the rate of other indicators of asthma burden in specific year were calculated in the same way.

Appendix 4: Methods of slope index of inequality and relative concentration index calculation

Slope index of inequality

We calculated the slope index of inequality (SII) and the relative concentration index (RCI) using methods defined by the Health Equity Assessment Toolkit (HEAT and HEAT Plus) version 5.0. Details were available in the technical note of the Health Equity Assessment Toolkit[4]. Within our research, the SII served as an absolute measure of inequality. It quantifies the disparity between countries characterized by the greatest and least asthma burden. Calculation of SII involved the utilization of suitable regression models, while considering all population subgroups. The SII was computed based on SDI level (Socio-Demographic Index). A representative sample of the entire population was assigned weights and ranked in rank order, from the country with the lowest SDI (rank 0) to the country with the highest SDI (rank 1). The ranking procedure took into account the proportional distribution of the population within each subgroup. The population within each country was then evaluated based on its position in the cumulative population distribution and the corresponding midpoint. A regression analysis of asthma burden was conducted against the midpoint values using the Health Economic Assessment Tool (HEAT), following the current methodology defined in GBD 2021. The analysis used a generalized linear model with a logit link function to facilitate the calculation of predictor values at the two extremes (rank 1 and rank 0). For asthma burden, SII value was the difference between the rank 0 (v_0) and rank 1 (v_1):

$$\text{SII} = v_0 - v_1$$

Greater absolute value of SII indicated higher inequality. A positive value indicates that the indicator was concentrated in countries with high SDI, while a negative value indicates that the indicator was concentrated in countries with low SDI.

Relative concentration index

The RCI, which represented for relative inequality indicator, was computed for 204 countries in this study, utilizing SDI levels as the basis. It provided an assessment of the degree to which the asthma burden was concentrated within subpopulations characterized by low or high SDIs. Each subgroup was assigned a weight proportional to its share of the population, ensuring proper consideration of their significance in the analysis. The RCI was calculated by dividing the absolute concentration index (ACI) by the set mean μ and multiplying by 100. ACI was calculated according to SDI level, ranking the entire population from the country with the lowest SDI level (rank 0) to the highest SDI level (rank 1). The formula for calculating the relative rank of a country was $X_{j=} \sum_j p_j - 0.5p_j$, so the formula for ACI was ACI=$\sum_j p_j \left(2x_j - 1\right)y_j$. Thus,

$$\text{RCI} = \frac{\sum_j p_j (2x_j - 1) y_j}{\mu} * 100$$

The RCI ranged between −100 and +100, with greater the absolute value indicated higher inequality. A positive value indicates that RCI was concentrated in countries with high SDI levels, while a negative value indicates that RCI was concentrated in countries with low SDI levels.

Appendix 5: Code for health inequality analysis

The following is an example code for the health inequality analysis:

```
library(mgcv)
library(splines)
library(broom)
# Data preparation -------------------------------------------------------
burden <- read.csv("asthma.csv", header = T)
burden <- burden %>%
```

```
  filter(age == "All ages",
         measure == "Prevalence",
         sex == "Both")
sdi <- read.csv("各国各年份 SDI(已校对).csv", header = T, check.names = F)
sdi <- sdi %>%
  pivot_longer(cols = `1990`:`2019`, names_to = "year") %>%
  rename(sdi = value) %>%
  dplyr::select(location, year, sdi)
sdi$year <- as.integer(sdi$year)
data <- left_join(burden, sdi, by = c("location", "year"))
files <- list.files(dir,
                    pattern = ".CSV",
                    full.names = T)
pop <- map_dfr(files, read.csv)
pop <- pop %>%
  dplyr::select(location_name, sex_name, age_group_name, year_id, metric_name, val)
colnames(pop) <- c("location", "sex", "age", "year", "metric", "val")
pop$sex[pop$sex == "male"] <- "Male"
pop$sex[pop$sex == "female"] <- "Female"
pop$sex[pop$sex == "both"] <- "Both"
pop <- pop %>%
  filter(age == "All Ages") %>%
  dplyr::select("location", "sex", "year", "val") %>%
  rename(pop = val)
mydata <- left_join(data, pop,
                    by = c("location", "sex", "year"))
# Slope index of inequality visualization ---------------------------------
## 1. Prepare data for plotting ------------------------------------------
a <- mydata %>%
```

```
  filter(metric == "Number") %>%
  group_by(year) %>%
  summarise(sum = sum(pop))
pop1990 <- a$sum[1]
pop2019 <- a$sum[2]
rank <- mydata %>%
  mutate(pop_global = ifelse(year == 1990, pop1990, pop2019)) %>%
  group_by(year, metric) %>%
  arrange(sdi) %>%
  mutate(cummu = cumsum(pop)) %>%
  mutate(half = pop / 2) %>%
  mutate(midpoint = cummu - half) %>%
  mutate(weighted_order = midpoint / pop_global)
rank$year <- factor(rank$year)
temp1 <- rank %>%
  filter(metric == "Rate") %>%
  filter(year == 1990)
temp2 <- rank %>%
  filter(metric == "Rate") %>%
  filter(year == 2019)
options(digits = 7)
fit1 <- lm(data = temp1, val ~ weighted_order)
fit2 <- lm(data = temp2, val ~ weighted_order)
ncvTest(fit1)
ncvTest(fit2)
r.huber1 <- rlm(data = temp1, val ~ weighted_order)
r.huber2 <- rlm(data = temp2, val ~ weighted_order)
coef(r.huber1)
coef(r.huber2)
confint.default(r.huber1)
```

```
confint.default(r.huber2)

# 2. Plotting -----------------------------------------------------------
color <- c("#0099B3", "#EC0000")
colnames(rank)
p1 <- rank %>%
  filter(metric == "Rate") %>%
  ggplot(aes(x = weighted_order, y = val, fill = year, group = year, color = year)) +
  geom_point(aes(color = year, size = pop / 1e6), alpha = 0.8, shape = 21) +
  scale_size_area("Population\n(million)", breaks = c(200, 400, 600, 800, 1000, 1200)) +
  geom_smooth(method = "rlm", size = 0.6, alpha = 0.1) +
  scale_fill_manual(values = color) +
  scale_color_manual(values = color) +
  geom_brace(aes(x = c(1.003, 1.103), y = c(754.6296, 754.6296 - 110.9277)),
             inherit.data = F, size = 0.6,
             rotate = 90, color = "#0099B3") +
  geom_brace(aes(c(1, 1.1), c(646.0465, 646.0465 - 179.5766)),
             inherit.data = F, size = 0.6,
             rotate = 90, color = "#EC0000") +
  geom_segment(x = 0.02, xend = 0.99,
               y = 754.6296, yend = 754.6296,
               color = "#0099B3", linetype = 2, size = 0.4, alpha = 0.4) +
  geom_segment(x = 0.02, xend = 0.99,
               y = 646.0465, yend = 646.0465,
               color = "#EC0000", linetype = 2, size = 0.4, alpha = 0.4)
p1 <- p1 + scale_x_continuous(limits = c(0, 1.22), labels = c("0", "0.25", "0.50", "0.75", "1.00",
"")) +
  xlab("Relative rank by SDI") +
  ylab("Crude Asthma Incidence rate (per 100,000)") +
  theme_bw() +
```

```
  theme(text = element_text(family = "serif"),
        plot.title = element_text(hjust = 0.5))
p1
# Concentration index visualization ---------------------------------------
# 1. Prepare data for plotting -------------------------------------------
a <- mydata %>%
  filter(metric == "Number") %>%
  group_by(year) %>%
  summarise(sum = sum(val))
daly1990 <- a$sum[1]
daly2019 <- a$sum[2]
ci <- rank %>%
  filter(metric == "Number") %>%
  mutate(total_daly = ifelse(year == 1990, daly1990, daly2019)) %>%
  group_by(year) %>%
  arrange(sdi) %>%
  mutate(cummu_daly = cumsum(val)) %>%
  mutate(frac_daly = cummu_daly / total_daly) %>%
  mutate(frac_population = cummu / pop_global)
# 2. Plotting -----------------------------------------------------------
p2 <- ci %>%
  ggplot(aes(x = frac_population, y = frac_daly, fill = year, color = year, group = year)) +
  geom_segment(x = 0, xend = 1,
               y = 0, yend = 0,
               linetype = 1, size = 1, color = "gray") +
  geom_segment(x = 1, xend = 1,
               y = 0, yend = 1,
               linetype = 1, size = 1, color = "gray") +
  geom_segment(x = 0, xend = 1,
               y = 0, yend = 1,
```

```
                    color = "#FFBC42", linetype = 1, size = 0.7, alpha = 1) +
  geom_point(aes(fill = year, size = pop / 1e6), alpha = 0.75, shape = 21) +
  scale_fill_manual(values = color) +
  scale_size_area("Population\n(million)", breaks = c(200, 400, 600, 800, 1000, 1200)) +
  geom_smooth(method = "gam",
              formula = y ~ ns(x,
                               knots = c(0.0000000001, 0.25, 0.5, 0.75, 0.9999999),
                               Boundary.knots = c(0, 1)),
              linetype = 1, size = 0.1, alpha = 0.6, se = T) +
  scale_color_manual(values = color)
p2 <- p2 + xlab("Cumulative fraction of population ranked by SDI") +
  ylab("Cumulative fraction of Incidence") +
  theme_bw() +
  theme(text = element_text(family = "serif"),
        plot.title = element_text(hjust = 0.5))
p2
```

References

1. GBD 2021 Diseases and Injuries Collaborators. Global incidence, prevalence, years lived with disability (YLDs), disability-adjusted life-years (DALYs), and healthy life expectancy (HALE) for 371 diseases and injuries in 204 countries and territories and 811 subnational locations, 1990-2021: a systematic analysis for the Global Burden of Disease Study 2021. *Lancet (London, England)* 2024; **403**(10440): 2133-2161.

2. Zhang S, Gao Z, Wu L, et al. Global patterns of asthma burden related to environmental risk factors during 1990-2019: an age-period-cohort analysis for global burden of disease study 2019. *Environmental health : a global access science source* 2024; **23**(1): 20.

3. Xie Y, Bowe B, Mokdad AH, et al. Analysis of the Global Burden of Disease study highlights the global, regional, and national trends of chronic kidney disease epidemiology from 1990 to 2016. *Kidney international* 2018; **94**(3): 567-581.

4. World Health Organization. *Health Equity Assessment Toolkit: Software for Exploring and Comparing Health Inequalities in Countries*. Version 5.0. Geneva: World Health Organization; 2023.

Table S1. Age-specific global asthma deaths rate attributed to risk factors in 1990 and 2021.

	Asthma deaths rate attributed to smoking (per 100,000)		Asthma deaths rate attributed to high body-mass index (per 100,000)		Asthma deaths rate attributed to occupational asthmagens (per 100,000)	
	1990	2021	1990	2021	1990	2021
**Both**						
Age-standardized	1.04 (0.12, 2.12)	0.10 (0.01, 0.19)	1.00 (0.43, 1.58)	0.74 (0.31, 1.19)	0.69 (0.54, 1.04)	0.35 (0.28, 0.50)
<5 years	-	-	0.06 (0.03, 0.11)	0.03 (0.01, 0.05)	-	-
5-14 years	-	-	0.02 (0.01, 0.04)	0.02 (0.01, 0.03)	-	-
15-49 years	0.20 (0.02, 0.38)	0.10 (0.01, 0.19)	0.20 (0.09, 0.33)	0.22 (0.10, 0.35)	0.31 (0.25, 0.38)	0.21 (0.17, 0.26)
50-69 years	2.80 (0.35, 5.71)	1.03 (0.12, 2.07)	2.26 (0.94, 3.63)	1.63 (0.68, 2.59)	2.39 (1.76, 3.67)	1.13 (0.88, 1.68)
≥70 years	7.81 (0.88, 16.19)	3.34 (0.37, 7.03)	8.16 (3.37, 12.80)	6.03 (2.50, 9.85)	2.63 (1.74, 4.71)	1.23 (0.87, 1.95)
**Male**						
Age-standardized	1.97 (0.23, 4.04)	0.73 (0.09, 1.46)	1.05 (0.47, 1.74)	0.66 (0.29, 1.09)	1.08 (0.78, 1.83)	0.49 (0.39, 0.79)
<5 years	-	-	0.06 (0.03, 0.10)	0.03 (0.01, 0.05)	-	-
5-14 years	-	-	0.02 (0.01, 0.04)	0.02 (0.01, 0.02)	-	-
15-49 years	0.33 (0.04, 0.62)	0.17 (0.02, 0.31)	0.18 (0.08, 0.28)	0.19 (0.08, 0.30)	0.41 (0.32, 0.57)	0.26 (0.21, 0.35)
50-69 years	4.81 (0.61, 9.79)	1.74 (0.21, 3.39)	2.26 (0.97, 3.81)	1.45 (0.63, 2.38)	3.67 (2.53, 6.21)	1.62 (1.22, 2.67)
≥70 years	15.63 (1.79, 32.87)	6.07 (0.70, 12.48)	8.66 (3.75, 14.25)	5.32 (2.27, 8.79)	4.88 (2.87, 9.86)	1.97 (1.32, 3.74)
**Female**						
Age-standardized	0.32 (0.03, 0.67)	0.14 (0.01, 0.31)	0.98 (0.42, 1.61)	0.80 (0.35, 1.31)	0.35 (0.24, 0.51)	0.22 (0.16, 0.31)
<5 years	-	-	0.06 (0.02, 0.12)	0.03 (0.01, 0.05)	-	-
5-14 years	-	-	0.02 (0.01, 0.04)	0.02 (0.01, 0.03)	-	-
15-49 years	0.07 (0.01, 0.14)	0.03 (0.00, 0.06)	0.23 (0.10, 0.38)	0.26 (0.11, 0.42)	0.20 (0.14, 0.27)	0.15 (0.11, 0.21)
50-69 years	0.85 (0.09, 1.8)	0.36 (0.04, 0.79)	2.25 (0.94, 3.74)	1.80 (0.76, 2.96)	1.13 (0.72, 1.72)	0.66 (0.46, 0.98)
≥70 years	2.39 (0.23, 5.28)	1.21 (0.12, 2.71)	7.82 (3.27, 12.98)	6.58 (2.75, 11.01)	1.07 (0.56, 1.94)	0.64 (0.38, 1.08)

Table S2. Age distribution of global asthma DALYs rate attributed to risk factors in 1990 and 2021.

	Asthma DALYs rate attributed to smoking		Asthma DALYs rate attributed to high body-mass index		Asthma DALYs rate attributed to occupational asthmagens	
	1990	2021	1990	2021	1990	2021
**Both**						
Age-standardized	41.95 (5.00, 79.54)	16.19 (1.89, 31.74)	50.53 (24.72, 78.10)	39.42 (19.62, 60.16)	38.05 (30.56, 48.67)	20.75 (16.70, 26.50)
<5 years	-	-	15.97 (7.18, 28.38)	15.38 (6.86, 27.96)	-	-
5-14 years	-	-	13.63 (6.17, 24.88)	17.99 (8.23, 33.06)	-	-
15-49 years	21.48 (2.76, 40.76)	10.55 (1.32, 20.34)	28.37 (12.62, 46.38)	28.84 (13.22, 45.73)	35.47 (27.21, 45.28)	23.11 (18.04, 29.58)
50-69 years	126.28 (14.92, 240.17)	46.01 (5.25, 87.50)	123.13 (54.81, 191.68)	80.91 (35.44, 125.93)	102.57 (79.46, 141.17)	49.95 (40.19, 67.14)
≥70 years	165.34 (18.10, 325.91)	65.43 (7.26, 134.38)	194.97 (82.26, 313.28)	128.91 (54.45, 205.96)	57.19 (38.44, 95.71)	26.56 (19.38, 39.96)
**Male**						
Age-standardized	30.57 (3.95, 56.60)	25.63 (3.02, 48.39)	47.08 (23.02, 73.37)	34.51 (17.82, 52.74)	52.58 (41.00, 74.32)	26.25 (20.96, 35.99)
<5 years	-	-	17.13 (7.76, 30.60)	16.95 (7.56, 31.06)	-	-
5-14 years	-	-	14.12 (6.42, 25.69)	18.99 (8.65, 34.92)	-	-
15-49 years	200.70 (24.24, 383.29)	15.33 (1.91, 29.35)	24.04 (11.10, 38.88)	23.63 (10.90, 37.64)	44.08 (33.40, 57.16)	26.94 (20.67, 35.75)
50-69 years	320.63 (35.74, 647.15)	71.63 (8.33, 132.74)	111.74 (49.84, 174.01)	68.52 (30.41, 108.69)	150.54 (109.03, 226.02)	67.57 (52.39, 100.6)
≥70 years	68.40 (8.20, 128.69)	116.02 (13.27, 232.31)	199.18 (86.56, 324.82)	113.88 (48.82, 181.80)	104.62 (65.55, 199.25)	42.15 (29.00, 72.41)
**Female**						
Age-standardized	18.85 (2.13, 38.26)	7.59 (0.82, 15.67)	54.23 (26.62, 85.11)	44.11 (21.42, 68.29)	24.29 (17.64, 31.61)	15.46 (11.77, 19.71)
<5 years	-	-	14.73 (6.38, 26.95)	13.71 (6.23, 25.38)	-	-
5-14 years	-	-	13.11 (5.86, 23.80)	16.92 (7.73, 30.85)	-	-
15-49 years	12.14 (1.45, 24.96)	21.38 (2.21, 44.77)	32.81 (14.39, 53.79)	34.19 (15.66, 53.96)	26.64 (19.03, 35.96)	19.19 (14.04, 25.24)
50-69 years	53.83 (5.85, 111.7)	25.71 (2.69, 56.68)	134.21 (60.77, 214.52)	92.83 (39.99, 145.46)	55.88 (38.27, 79.55)	33.02 (23.92, 44.22)
≥70 years	57.67 (6.00, 118.79)	5.65 (0.61, 11.82)	192.04 (80.94, 314.22)	140.71 (60.04, 228.19)	24.30 (14.12, 40.70)	14.32 (8.96, 22.28)

Abbreviations: DALYs, disability-adjusted life-years;

Table S3: Changes in asthma burden number according to population-level determinants and causes from 1990 to 2021.

GBD data	Change due to Population-level determinants (% contribute to the total changes)			
	Overall difference [a]	Aging [b]	Population [c]	Epidemiological change [d]
**Both**				
Incidence				
Global	-3691452.61	-5265749.85 (142.65)	16035381.08 (-434.39)	-14461083.84 (391.75)
High SDI	-690513.33	-836463.64 (121.14)	1627522.37 (-235.70)	-1481572.05 (214.56)
High-middle SDI	-1861766.62	-818950.16 (43.99)	1103633.59 (-59.28)	-2146450.05 (115.29)
Middle SDI	-2698477.35	-2441701.14 (90.48)	4129191.61 (-153.02)	-4385967.83 (162.53)
Low-middle SDI	-1128995.68	-1557365.2 (137.94)	4681880.20 (-414.69)	-4253510.68 (376.75)
Low SDI	2693835.08	-711305.75 (-26.40)	5553500.15 (206.16)	-2148359.32 (-79.75)
Prevalence				
Global	-26821701.17	4480506.05 (-16.70)	112106340.76 (-417.97)	-143408547.98 (534.67)
High SDI	-13421608.07	3874894.97 (-28.87)	17618840.66 (-131.27)	-34915343.70 (260.14)
High-middle SDI	-14550526.64	2368625.07 (-16.28)	8957328.33 (-61.56)	-25876480.04 (177.84)
Middle SDI	-10172749.66	-3854932.48 (37.89)	24368479.97 (-239.55)	-30686297.14 (301.65)
Low-middle SDI	-2531726.19	-2063068.61 (81.49)	26689420.44 (-1054.2)	-27158078.02 (1072.71)
Low SDI	13925635.16	-1550878.37 (-11.14)	29590252.8 (212.49)	-14113739.28 (-101.35)
Deaths				
Global	59649.62	76042.52 (127.48)	82906.59 (138.99)	-99299.48 (-166.47)
High SDI	-9289.51	7360.04 (-79.23)	3615.37 (-38.92)	-20264.92 (218.15)
High-middle SDI	-5856.38	8859.26 (-151.28)	3454.18 (-58.98)	-18169.82 (310.26)
Middle SDI	13557.32	35260.27 (260.08)	20079.52 (148.11)	-41782.47 (-308.19)
Low-middle SDI	46002.93	35732.78 (77.68)	44219.11 (96.12)	-33948.96 (-73.80)
Low SDI	15227.76	2166.86 (14.23)	29678.91 (194.90)	-16618.01 (-109.13)
DALYs				
Global	-1439189.80	2966644.95 (-206.13)	9091014.68 (-631.68)	-13496849.43 (937.81)
High SDI	-1132131.01	325299.67 (-28.73)	849736.62 (-75.06)	-2307167.29 (203.79)
High-middle SDI	-1120385.72	378657.25 (-33.80)	526329.13 (-46.98)	-2025372.1 (180.77)
Middle SDI	-570746.35	1061992.79 (-186.07)	2055151.05 (-360.08)	-3687890.19 (646.15)
Low-middle SDI	553023.24	1217697.70 (220.19)	3562505.39 (644.19)	-4227179.85 (-764.38)
Low SDI	835802.68	-68920.82 (-8.25)	3127256.43 (374.16)	-2222532.94 (-265.92)
**Male**				

Incidence				
Global	-2450639.84	-2967887.49 (121.11)	8235709.57 (-336.06)	-7718461.92 (314.96)
High SDI	-437785.37	-498660.69 (113.91)	821632.27 (-187.68)	-760756.94 (173.77)
High-middle SDI	-942742.83	-466897.19 (49.53)	579395.23 (-61.46)	-1055240.88 (111.93)
Middle SDI	-1481236.9	-1329395.31 (89.75)	2142383.27 (-144.63)	-2294224.85 (154.89)
Low-middle SDI	-882215.44	-896278.74 (101.59)	2446844.09 (-277.35)	-2432780.8 (275.76)
Low SDI	1296239.39	-380981.95 (-29.39)	2854051.68 (220.18)	-1176830.35 (-90.79)
Prevalence				
Global	-17326389.52	-305353.20 (1.76)	54262490.87 (-313.18)	-71283527.19 (411.42)
High SDI	-8078878.51	1454527.49 (-18.00)	8217167.06 (-101.71)	-17750573.06 (219.72)
High-middle SDI	-7180897.54	760172.00 (-10.59)	4383088.41 (-61.04)	-12324157.96 (171.62)
Middle SDI	-5299700.3	-2527434.30 (47.69)	12240075.31 (-230.96)	-15012341.32 (283.27)
Low-middle SDI	-3161833.85	-1636675.67 (51.76)	13706900.56 (-433.51)	-15232058.74 (481.75)
Low SDI	6430041.37	-891454.81 (-13.86)	14767928.79 (229.67)	-7446432.61 (-115.81)
Deaths				
Global	2166.08	89997.34 (4154.85)	84026.01 (3879.17)	-171857.27 (-7934.02)
High SDI	-13907.34	9079.42 (-65.29)	3950.52 (-28.41)	-26937.28 (193.69)
High-middle SDI	-8791.25	10840.35 (-123.31)	3834.03 (-43.61)	-23465.64 (266.92)
Middle SDI	4660.34	36964.59 (793.17)	20022.24 (429.63)	-52326.49 (-1122.80)
Low-middle SDI	16506	28762.37 (174.25)	44119.21 (267.29)	-56375.58 (-341.55)
Low SDI	3742.67	-56.47 (-1.51)	27251.82 (728.14)	-23452.68 (-626.63)
DALYs				
Global	-1569925.09	1563804.62 (-99.61)	4552283.69 (-289.97)	-7686013.40 (489.58)
High SDI	-671467.91	169064.93 (-25.18)	414543.96 (-61.74)	-1255076.80 (186.92)
High-middle SDI	-598709.8	204866.47 (-34.22)	269716.75 (-45.05)	-1073293.02 (179.27)
Middle SDI	-394576.72	547152.95 (-138.67)	1040028.37 (-263.58)	-1981758.04 (502.25)
Low-middle SDI	-139240.14	529318.82 (-380.15)	1816643.03 (-1304.68)	-2485201.98 (1784.83)
Low SDI	237108.3	-62406.41 (-26.32)	1541328.90 (650.05)	-1241814.20 (-523.73)
**Female**				
Incidence				
Global	-1240812.77	-2276896.83 (183.50)	7795811.67 (-628.28)	-6759727.6 (544.78)
High SDI	-252727.96	-344380.18 (136.27)	803503.07 (-317.93)	-711850.85 (281.67)
High-middle SDI	-919023.79	-349496.10 (38.03)	525077.83 (-57.13)	-1094605.52 (119.11)
Middle SDI	-1217240.46	-1099023.27 (90.29)	1979219.59 (-162.60)	-2097436.78 (172.31)
Low-middle SDI	-246780.24	-654481.38 (265.21)	2226734.79 (-902.31)	-1819033.66 (737.11)

Low SDI	1397595.70	-328729.15 (-23.52)	2697751.00 (193.03)	-971426.15 (-69.51)
Prevalence				
Global	-9495311.64	4978439.33 (-52.43)	57892568.06 (-609.70)	-72366319.03 (762.13)
High SDI	-5342729.56	2368290.45 (-44.33)	9308084.63 (-174.22)	-17019104.63 (318.55)
High-middle SDI	-7369629.1	1654289.59 (-22.45)	4567030.24 (-61.97)	-13590948.93 (184.42)
Middle SDI	-4873049.35	-1226890.12 (25.18)	12113271.54 (-248.58)	-15759430.78 (323.40)
Low-middle SDI	630107.64	-393387.99 (-62.43)	12947983.74 (2054.88)	-11924488.1 (-1892.45)
Low SDI	7495593.79	-649263.17 (-8.66)	14820204.08 (197.72)	-6675347.12 (-89.06)
Deaths				
Global	59649.62	76042.52 (127.48)	82906.59 (138.99)	-99299.48 (-166.47)
High SDI	-9289.51	7360.04 (-79.23)	3615.37 (-38.92)	-20264.92 (218.15)
High-middle SDI	-5856.38	8859.26 (-151.28)	3454.18 (-58.98)	-18169.82 (310.26)
Middle SDI	13557.32	35260.27 (260.08)	20079.52 (148.11)	-41782.47 (-308.19)
Low-middle SDI	46002.93	35732.78 (77.68)	44219.11 (96.12)	-33948.96 (-73.80)
Low SDI	15227.76	2166.86 (14.23)	29678.91 (194.90)	-16618.01 (-109.13)
DALYs				
Global	130735.29	1454750.18 (1112.74)	4544135.93 (3475.83)	-5868150.82 (-4488.57)
High SDI	-460663.09	166680.58 (-36.18)	433457.93 (-94.09)	-1060801.6 (230.28)
High-middle SDI	-521675.93	186713.42 (-35.79)	257413.38 (-49.34)	-965802.74 (185.13)
Middle SDI	-176169.63	520300.7 (-295.34)	1013173.10 (-575.11)	-1709643.43 (970.45)
Low-middle SDI	692263.37	677236.75 (97.83)	1739148.12 (251.23)	-1724121.49 (-249.06)
Low SDI	598694.38	-4723.48 (-0.79)	1585358.92 (264.80)	-981941.06 (-164.01)

Note:

a.Change in case number between year 2021 and 1990

b.Change in case number due to change in the age structure

c.Change in case number due to change in population number

d.Change in case number due to epidemiologic changes. Epidemiologic changes refer to the case number change when age structure and population hold constant

Table S4: Changes in asthma burden attribute to smoking according to population-level determinants of population growth, aging, and epidemiological change from 1990 to 2021.

GBD data	Change due to Population-level determinants (% contribute to the total changes)			
	Overall difference [a]	Aging [b]	Population [c]	Epidemiological change [d]
**Both**				
Deaths				
Global	-5062.65	6566.75 (-129.71)	27165.00 (-536.58)	-38794.40 (766.29)
High SDI	-3746.24	883.53 (-23.58)	1610.53 (-42.99)	-6240.30 (166.57)
High-middle SDI	-2331.14	803.81 (-34.48)	2089.20 (-89.62)	-5224.15 (224.10)
Middle SDI	-890.36	3162.34 (-355.18)	8900.05 (-999.60)	-12952.74 (1454.78)
Low-middle SDI	1649.24	2061.64 (125.01)	14286.20 (866.23)	-14698.60 (-891.23)
Low SDI	267.32	-175.87 (-65.79)	4069.89 (1522.48)	-3626.71 (-1356.69)
DALYs				
Global	-376428.68	130563.48 (-34.68)	1146579.93 (-304.59)	-1653572.08 (439.28)
High SDI	-226619.47	13526.07 (-5.97)	158427.19 (-69.91)	-398572.73 (175.88)
High-middle SDI	-127487.00	15020.99 (-11.78)	126862.61 (-99.51)	-269370.61 (211.29)
Middle SDI	-35620.16	58259.56 (-163.56)	314935.70 (-884.15)	-408815.42 (1147.71)
Low-middle SDI	7801.65	37424.82 (479.7)	438765.63 (5624.01)	-468388.80 (-6003.71)
Low SDI	6295.98	-5683.89 (-90.28)	134919.91 (2142.95)	-122940.04 (-1952.68)
**Male**				
Deaths				
Global	-4937.23	6816.40 (-138.06)	22457.18 (-454.85)	-34210.81 (692.91)
High SDI	-2859.39	894.14 (-31.27)	1247.61 (-43.63)	-5001.14 (174.90)
High-middle SDI	-2000.55	899.97 (-44.99)	1830.95 (-91.52)	-4731.47 (236.51)
Middle SDI	-723.16	2891.40 (-399.83)	7359.89 (-1017.74)	-10974.45 (1517.57)
Low-middle SDI	647.56	1323.14 (204.33)	11614.93 (1793.65)	-12290.51 (-1897.97)
Low SDI	8.06	-190.73 (-2366.38)	3005.00 (37282.82)	-2806.21 (-34816.45)
DALYs				
Global	-376428.68	130563.48 (-34.68)	1146579.93 (-304.59)	-1653572.08 (439.28)
High SDI	-226619.47	13526.07 (-5.97)	158427.19 (-69.91)	-398572.73 (175.88)
High-middle SDI	-127487.00	15020.99 (-11.78)	126862.61 (-99.51)	-269370.61 (211.29)
Middle SDI	-35620.16	58259.56 (-163.56)	314935.70 (-884.15)	-408815.42 (1147.71)
Low-middle SDI	7801.65	37424.82 (479.7)	438765.63 (5624.01)	-468388.80 (-6003.71)
Low SDI	6295.98	-5683.89 (-90.28)	134919.91 (2142.95)	-122940.04 (-1952.68)

**Female**				
Deaths				
Global	-125.42	1049.12 (-836.49)	4680.95 (-3732.22)	-5855.50 (4668.71)
High SDI	-886.85	226.13 (-25.50)	450.58 (-50.81)	-1563.56 (176.3)
High-middle SDI	-330.59	125.00 (-37.81)	323.03 (-97.71)	-778.62 (235.52)
Middle SDI	-167.20	468.47 (-280.19)	1295.32 (-774.71)	-1930.99 (1154.90)
Low-middle SDI	1001.69	395.95 (39.53)	2145.65 (214.20)	-1539.91 (-153.73)
Low SDI	259.26	-19.43 (-7.50)	1002.13 (386.54)	-723.44 (-279.04)
DALYs				
Global	-79382.19	21192.47 (-26.70)	272751.71 (-343.59)	-373326.37 (470.29)
High SDI	-69946.01	511.81 (-0.73)	65316.93 (-93.38)	-135774.75 (194.11)
High-middle SDI	-22686.37	1406.45 (-6.20)	30475.30 (-134.33)	-54568.11 (240.53)
Middle SDI	-10347.43	7960.55 (-76.93)	47172.73 (-455.89)	-65480.71 (632.82)
Low-middle SDI	18260.99	7091.84 (38.84)	64359.57 (352.44)	-53190.42 (-291.28)
Low SDI	5583.55	-919.45 (-16.47)	31446.19 (563.19)	-24943.20 (-446.73)

Note:

a.Change in case number between year 2021 and 1990

b.Change in case number due to change in the age structure

c.Change in case number due to change in population number

d.Change in case number due to epidemiologic changes. Epidemiologic changes refer to the case number change when age structure and population hold constant

DALYs: Disability-Adjusted Life Years; SDI: Socio-demographic index.

Table S5: Changes in asthma burden attribute to high BMI according to population-level determinants of population growth, aging, and epidemiological change from 1990 to 2021.

GBD data	Change due to Population-level determinants (% contribute to the total changes)			
	Overall difference [a]	Aging [b]	Population [c]	Epidemiological change [d]
**Both**				
Deaths				
Global	24322.67	20360.69 (83.71)	19663.59 (80.84)	-15701.60 (-64.56)
High SDI	-3099.46	3016.79 (-97.33)	1512.01 (-48.78)	-7628.25 (246.12)
High-middle SDI	-1396.81	3747.26 (-268.27)	1396.28 (-99.96)	-6540.35 (468.23)
Middle SDI	9346.55	9086.66 (97.22)	4973.75 (53.21)	-4713.87 (-50.43)
Low-middle SDI	14711.49	6124.03 (41.63)	8041.27 (54.66)	546.18 (3.71)
Low SDI	4758.20	214.87 (4.52)	4902.33 (103.03)	-358.99 (-7.54)
DALYs				
Global	1029358.36	622035.94 (60.43)	1091841.98 (106.07)	-684519.56 (-66.50)
High SDI	18106.51	114965.83 (634.94)	172909.54 (954.96)	-269768.86 (-1489.90)
High-middle SDI	-55598.37	130242.56 (-234.26)	91481.20 (-164.54)	-277322.12 (498.80)
Middle SDI	364396.37	215087.04 (59.03)	226260.94 (62.09)	-76951.61 (-21.12)
Low-middle SDI	477569.44	152239.16 (31.88)	301902.34 (63.22)	23427.94 (4.91)
Low SDI	224779.54	3675.66 (1.64)	223663.51 (99.5)	-2559.64 (-1.14)
**Male**				
Deaths				
Global	8231.18	9610.72 (116.76)	8596.53 (104.44)	-9976.07 (-121.2)
High SDI	-2028.26	1643.85 (-81.05)	757.61 (-37.35)	-4429.72 (218.4)
High-middle SDI	-919.03	1828.84 (-199.00)	642.76 (-69.94)	-3390.62 (368.93)
Middle SDI	3721.92	4072.34 (109.41)	2181.58 (58.61)	-2531.99 (-68.03)
Low-middle SDI	5687.40	2304.04 (40.51)	3363.89 (59.15)	19.47 (0.34)
Low SDI	1773.45	11.49 (0.65)	1955.98 (110.29)	-194.02 (-10.94)
DALYs				
Global	386221.59	269750.74 (69.84)	473222.70 (122.53)	-356751.86 (-92.37)
High SDI	-28925.80	54717.69 (-189.17)	79342.47 (-274.30)	-162985.96 (563.46)
High-middle SDI	-29770.56	57442.99 (-192.95)	41263.19 (-138.60)	-128476.73 (431.56)
Middle SDI	160034.36	92978.18 (58.10)	100384.22 (62.73)	-33328.04 (-20.83)
Low-middle SDI	193398.01	56252.63 (29.09)	127631.83 (65.99)	9513.56 (4.92)
Low SDI	91610.85	-1111.86 (-1.21)	90325.70 (98.60)	2397.01 (2.62)

**Female**				
Deaths				
Global	16091.49	10821.85 (67.25)	11109.89 (69.04)	-5840.25 (-36.29)
High SDI	-1071.19	1489.40 (-139.04)	761.07 (-71.05)	-3321.66 (310.09)
High-middle SDI	-477.78	1969.34 (-412.19)	753.24 (-157.65)	-3200.36 (669.84)
Middle SDI	5624.62	5013.34 (89.13)	2808.74 (49.94)	-2197.46 (-39.07)
Low-middle SDI	9024.09	3885.91 (43.06)	4708.08 (52.17)	430.10 (4.77)
Low SDI	2984.75	236.19 (7.91)	2953.76 (98.96)	-205.20 (-6.88)
DALYs				
Global	643136.77	352784.49 (54.85)	620260.39 (96.44)	-329908.11 (-51.30)
High SDI	47032.31	60157.40 (127.91)	92610.99 (196.91)	-105736.07 (-224.82)
High-middle SDI	-25827.81	73042.77 (-282.81)	49968.34 (-193.47)	-148838.92 (576.27)
Middle SDI	204362.01	122676.77 (60.03)	126588.07 (61.94)	-44902.83 (-21.97)
Low-middle SDI	284171.43	97689.01 (34.38)	175382.76 (61.72)	11099.66 (3.91)
Low SDI	133168.68	5742.84 (4.31)	133674.62 (100.38)	-6248.78 (-4.69)

Note:

a.Change in case number between year 2021 and 1990

b.Change in case number due to change in the age structure

c.Change in case number due to change in population number

d.Change in case number due to epidemiologic changes. Epidemiologic changes refer to the case number change when age structure and population hold constant

DALYs: Disability-Adjusted Life Years; SDI: Socio-demographic index; BMI: Body Mass Index.

Table S6: Changes in asthma burden attribute to occupational asthmagen according to population-level determinants of population growth, aging, and epidemiological change from 1990 to 2021.

GBD data	Change due to Population-level determinants (% contribute to the total changes)			
	Overall difference [a]	Aging [b]	Population [c]	Epidemiological change [d]
**Both**				
Deaths				
Global	588.25	6871.07 (1168.05)	15869.52 (2697.75)	-22152.34 (-3765.80)
High SDI	-1274.20	240.28 (-18.86)	452.62 (-35.52)	-1967.10 (154.38)
High-middle SDI	-1258.47	555.27 (-44.12)	631.58 (-50.19)	-2445.32 (194.31)
Middle SDI	239.45	2930.86 (1224.00)	3958.93 (1653.34)	-6650.34 (-2777.34)
Low-middle SDI	2158.59	2346.74 (108.72)	9547.13 (442.29)	-9735.28 (-451.00)
Low SDI	720.20	-364.44 (-50.60)	6066.84 (842.38)	-4982.20 (-691.78)
DALYs				
Global	-15042.26	196093.02 (-1303.61)	919900.52 (-6115.44)	-1131035.80 (7519.05)
High SDI	-98273.69	-5929.52 (6.03)	89492.31 (-91.06)	-181836.48 (185.03)
High-middle SDI	-86914.43	16598.64 (-19.10)	62442.97 (-71.84)	-165956.04 (190.94)
Middle SDI	13628.99	82078.86 (602.24)	220789.46 (1620.00)	-289239.34 (-2122.24)
Low-middle SDI	87894.01	67466.01 (76.76)	395671.19 (450.17)	-375243.19 (-426.93)
Low SDI	68692.83	-10049.26 (-14.63)	282287.88 (410.94)	-203545.79 (-296.31)
**Male**				
Deaths				
Global	-1160.64	5451.98 (-469.74)	11350.90 (-977.99)	-17963.51 (1547.72)
High SDI	-1003.60	226.95 (-22.61)	350.02 (-34.88)	-1580.57 (157.49)
High-middle SDI	-999.60	470.08 (-47.03)	468.93 (-46.91)	-1938.62 (193.94)
Middle SDI	-153.09	2163.23 (-1413.04)	2764.74 (-1805.96)	-5081.06 (3319.00)
Low-middle SDI	994.20	1471.99 (148.06)	6941.63 (698.21)	-7419.42 (-746.27)
Low SDI	1.82	-350.18 (-19240.77)	3910.39 (214856.37)	-3558.38 (-195515.55)
DALYs				
Global	-92097.41	149856.60 (-162.72)	601638.48 (-653.26)	-843592.49 (915.98)
High SDI	-82983.76	157.47 (-0.19)	55677.42 (-67.09)	-138818.65 (167.28)
High-middle SDI	-65735.54	14075.97 (-21.41)	40286.64 (-61.29)	-120098.15 (182.70)
Middle SDI	-1979.77	59292.02 (-2994.89)	143321.91 (-7239.32)	-204593.71 (10334.22)
Low-middle SDI	36594.71	42055.56 (114.92)	278518.83 (761.09)	-283979.67 (-776.01)
Low SDI	22148.58	-9647.42 (-43.56)	172520.39 (778.92)	-140724.39 (-635.37)

**Female**				
Deaths				
Global	1748.89	1713.58 (97.98)	4493.03 (256.91)	-4457.72 (-254.89)
High SDI	-270.60	49.33 (-18.23)	119.95 (-44.33)	-439.87 (162.55)
High-middle SDI	-258.87	131.17 (-50.67)	174.71 (-67.49)	-564.74 (218.16)
Middle SDI	392.54	773.29 (197.00)	1136.48 (289.52)	-1517.24 (-386.52)
Low-middle SDI	1164.39	639.97 (54.96)	2443.14 (209.82)	-1918.71 (-164.78)
Low SDI	718.38	-66.18 (-9.21)	2127.31 (296.13)	-1342.75 (-186.91)
DALYs				
Global	77055.15	51614.09 (66.98)	316440.68 (410.67)	-290999.62 (-377.65)
High SDI	-15289.92	-4871.55 (31.86)	35058.04 (-229.29)	-45476.41 (297.43)
High-middle SDI	-21178.88	3930.53 (-18.56)	22747.70 (-107.41)	-47857.12 (225.97)
Middle SDI	15608.76	22471.08 (143.96)	74906.74 (479.90)	-81769.06 (-523.87)
Low-middle SDI	51299.30	19046.92 (37.13)	111341.32 (217.04)	-79088.94 (-154.17)
Low SDI	46544.25	-1819.63 (-3.91)	108781.02 (233.72)	-60417.14 (-129.81)

Note:

a.Change in case number between year 2021 and 1990

b.Change in case number due to change in the age structure

c.Change in case number due to change in population number

d.Change in case number due to epidemiologic changes. Epidemiologic changes refer to the case number change when age structure and population hold constant

DALYs: Disability-Adjusted Life Years; SDI: Socio-demographic index.

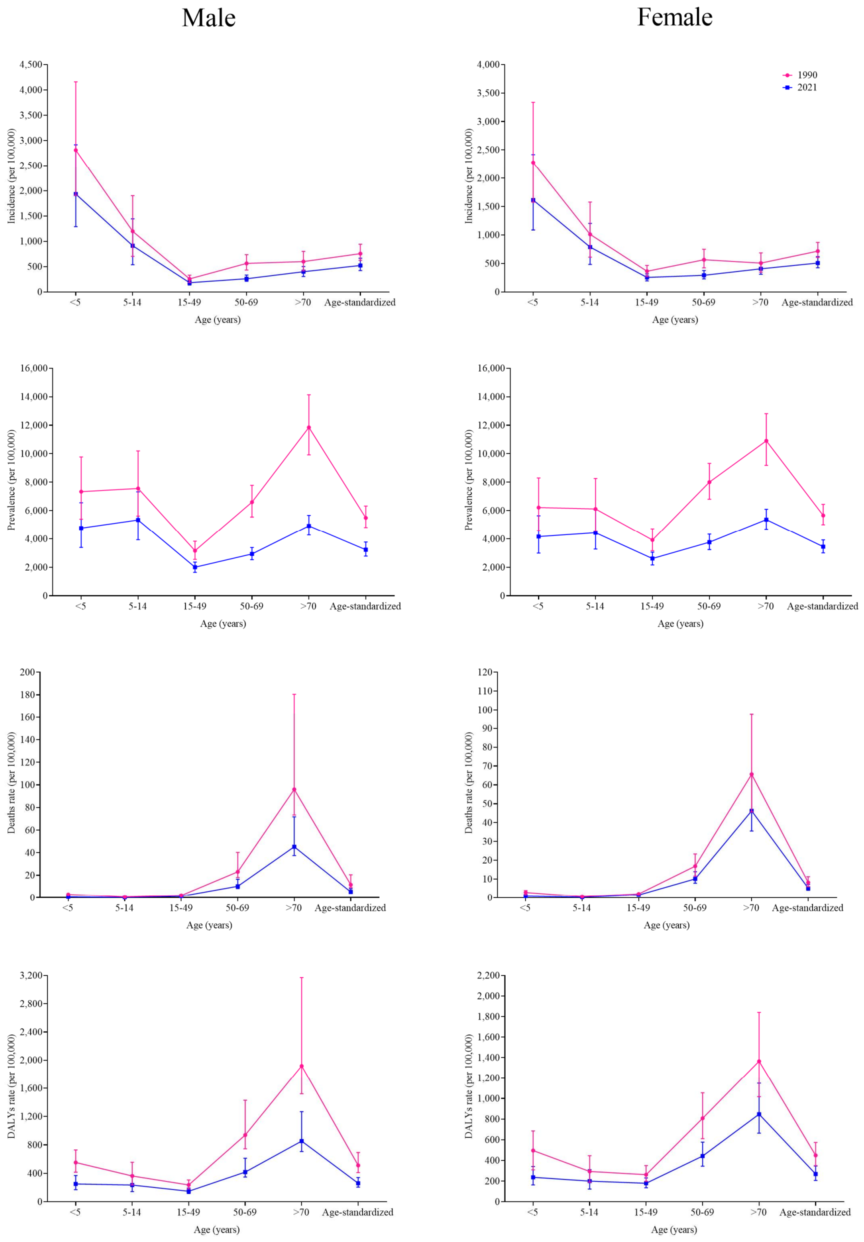

Figure S1 Age distribution of global asthma incidence, prevalence, deaths, and DALYs rates in 1990 and 2021, by sex.

Abbreviations: DALYs, disability-adjusted life-years.

Notes: A) Age distribution of asthma incidence in male, 1990 and 2021; B) Age distribution of asthma incidence in female, 1990 and 2021; C) Age distribution of asthma prevalence in male, 1990 and 2021; D) Age distribution of asthma prevalence in female, 1990 and 2021; E) Age distribution of asthma deaths rates in male, 1990 and 2021; F) Age distribution of asthma deaths rates in female, 1990 and 2021; G) Age distribution of DALYs rates in male, 1990 and 2021; H) Age distribution of asthma DALYs rates in female worldwide, 1990 and 2021.

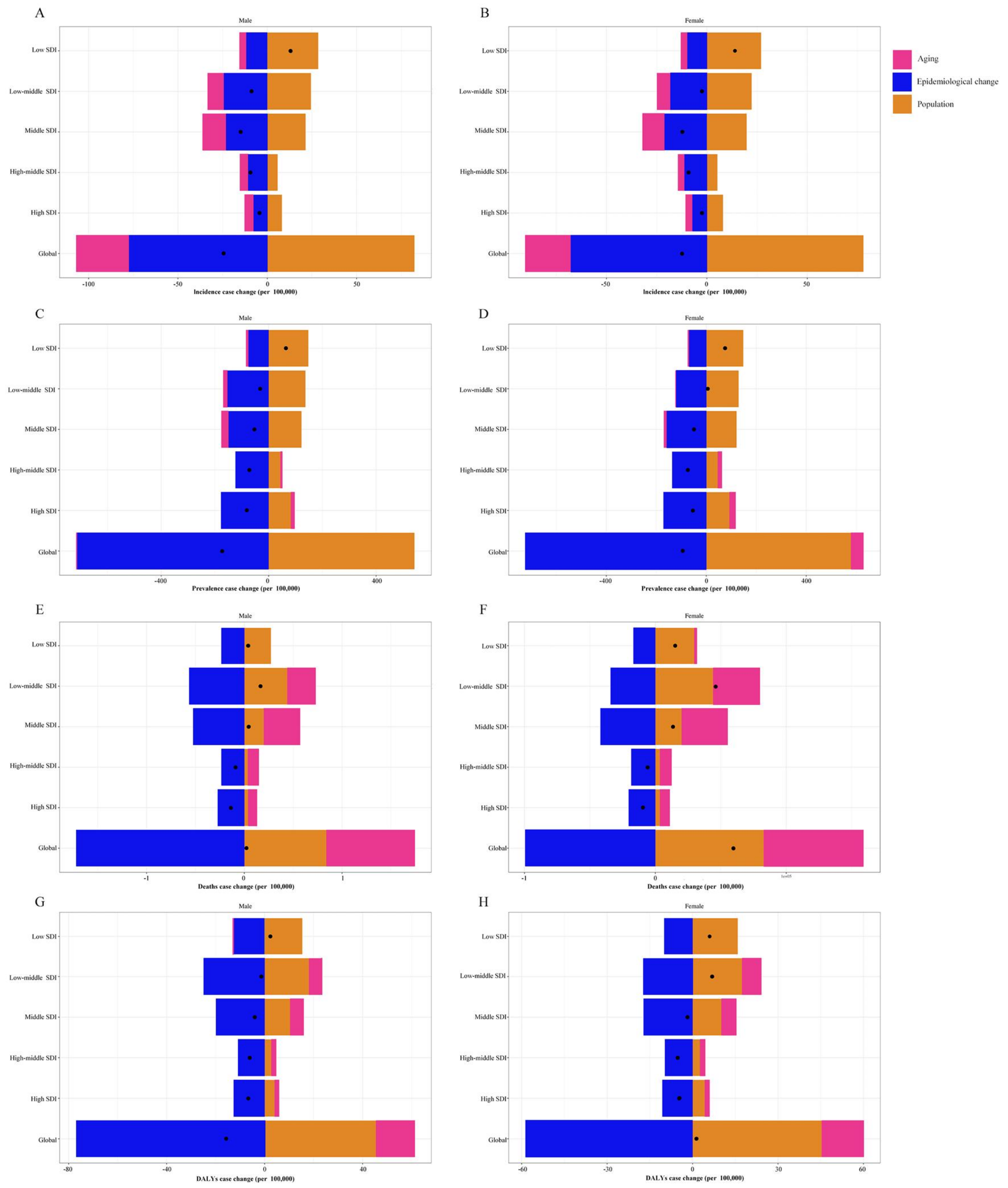

Figure S2 Decomposition analysis of global changes in incidence, prevalence, deaths, and DALYs case change of asthma according to population-level determinants during 1990-2021, by SDI quintile and sex.

Abbreviations: SDI, socio-demographic index; DALYs, disability-adjusted life-years.

Notes: A) Decomposition analysis of the global changes in the number of asthma incidence in male from 1990 to 2021 by SDI; B) Decomposition analysis of the global changes in the number of asthma incidence in female from 1990 to 2021 by SDI; C) Decomposition analysis of the global changes in the number of

asthma prevalence in male from 1990 to 2021 by SDI; D) Decomposition analysis of the global changes in the number of asthma prevalence in female from 1990 to 2021 by SDI; E) Decomposition analysis of the global changes in the number of asthma deaths in male from 1990 to 2021 by SDI; F) Decomposition analysis of the global changes in the number of asthma deaths in female from 1990 to 2021 by SDI; G) Decomposition analysis of the global changes in the number of asthma DALYs in male from 1990 to 2021 by SDI; H) Decomposition analysis of the global changes in the number of asthma DALYs in female from 1990 to 2021 by SDI.

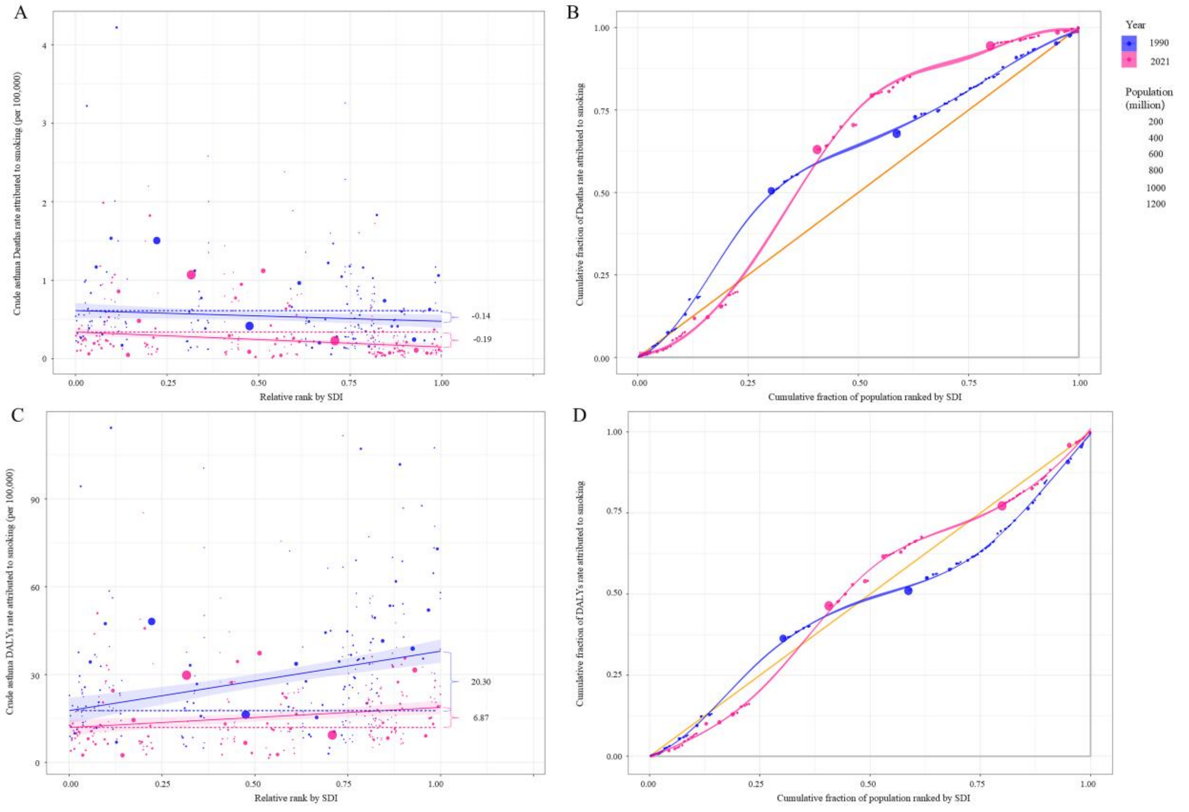

Figure S3 Health inequality regression curves and concentration curves for asthma deaths and DALYs rates attributed to smoking, 1990 and 2021.

Abbreviations: SII, Slope index of inequality; RCI, relative concentration index.

Notes: A) SII of asthma deaths rates attributed to smoking in 1990 and 2021; B) RCI of asthma deaths rates attributed to smoking in 1990 and 2021; C) SII of asthma DALYs rates attributed to smoking in 1990 and 2021; D) RCI of asthma DALYs rates attributed to smoking in 1990 and 2021.

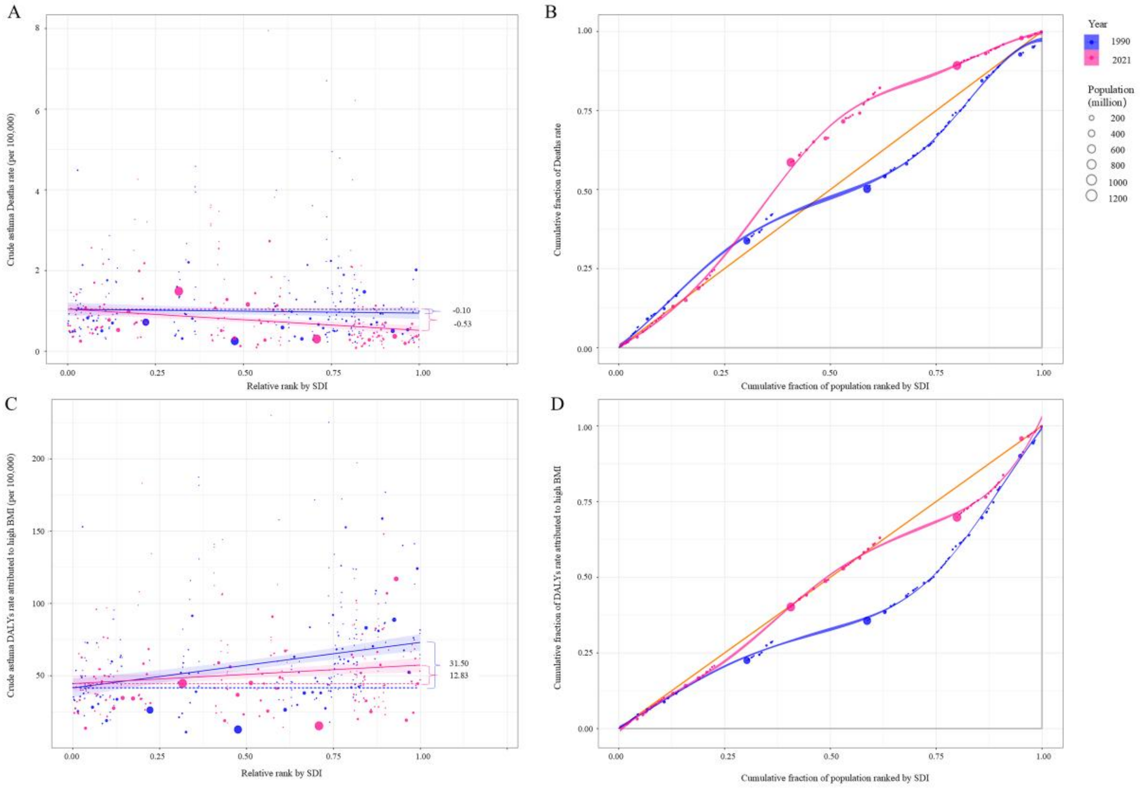

Figure S4 Health inequality regression curves and concentration curves for asthma deaths and DALYs rates attributed to high BMI, 1990 and 2021.

Abbreviations: BMI, body-mass index; SII, Slope index of inequality; RCI, relative concentration index.

Notes: A) SII of asthma deaths rates attributed to high BMI in 1990 and 2021; B) RCI of asthma deaths rates attributed to high BMI in 1990 and 2021; C) SII of asthma DALYs rates attributed to high BMI in 1990 and 2021; D) RCI of asthma DALYs rates attributed to high BMI in 1990 and 2021.

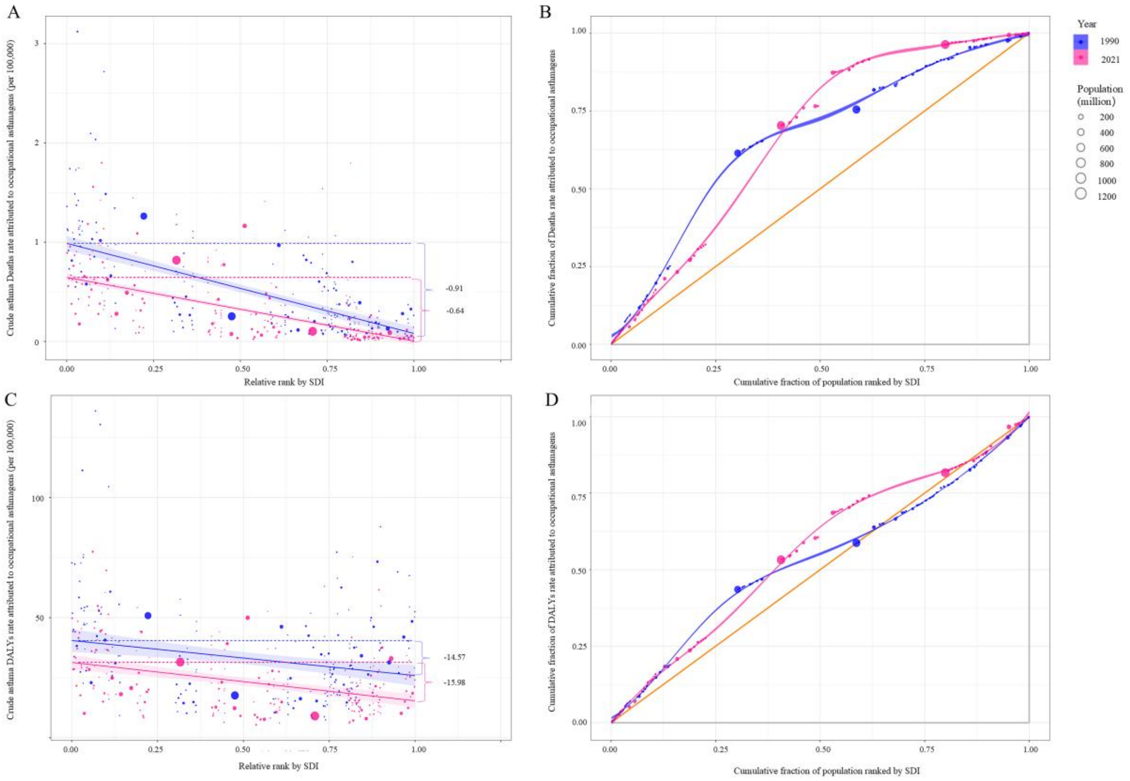

Figure S5 Health inequality regression curves and concentration curves for asthma deaths and DALYs rates attributed to occupational asthmagens, 1990 and 2021.

Abbreviations: SII, Slope index of inequality; RCI, relative concentration index.

Notes: A) SII of asthma deaths rates attributed to occupational asthmagens in 1990 and 2021; B) RCI of asthma deaths rates attributed to occupational asthmagens in 1990 and 2021; C) SII of asthma DALYs rates attributed to occupational asthmagens in 1990 and 2021; D) RCI of asthma DALYs rates attributed to occupational asthmagens in 1990 and 2021.
